# NERD-seq: a novel approach of Nanopore direct RNA sequencing that expands representation of non-coding RNAs

**DOI:** 10.1186/s13059-024-03375-8

**Published:** 2024-08-28

**Authors:** Luke Saville, Li Wu, Jemaneh Habtewold, Yubo Cheng, Babita Gollen, Liam Mitchell, Matthew Stuart-Edwards, Travis Haight, Majid Mohajerani, Athanasios Zovoilis

**Affiliations:** 1 Department of Biochemistry and Medical Genetics, University of Manitoba, Winnipeg, MB R3E3N4 Canada; 2 Paul Albrechtsen Research Institute, CCMB, Winnipeg, MB R3E3N4 Canada; 3 Southern Alberta Genome Sciences Centre, University of Lethbridge, Lethbridge, AB T1K3M4 Canada; 4 Canadian Centre for Behavioral Neuroscience, University of Lethbridge, Lethbridge, AB T1K3M4 Canada

## Abstract

Non-coding RNAs (ncRNAs) are frequently documented RNA modification substrates. Nanopore Technologies enables the direct sequencing of RNAs and the detection of modified nucleobases. Ordinarily, direct RNA sequencing uses polyadenylation selection, studying primarily mRNA gene expression. Here, we present NERD-seq, which enables detection of multiple non-coding RNAs, excluded by the standard approach, alongside natively polyadenylated transcripts. Using neural tissues as a proof of principle, we show that NERD-seq expands representation of frequently modified non-coding RNAs, such as snoRNAs, snRNAs, scRNAs, srpRNAs, tRNAs, and rRFs. NERD-seq represents an RNA-seq approach to simultaneously study mRNA and ncRNA epitranscriptomes in brain tissues and beyond.

**Supplementary Information:**

The online version contains supplementary material available at 10.1186/s13059-024-03375-8.

## Background

Since the development of RNA sequencing [1, 2], our understanding of the transcriptome and its regulation has grown substantially [3]. By expanding the context of transcriptome analysis, RNA sequencing allows for an improved understanding of the effects of cellular regulation [4, 5], the environment [6, 7], and disease pathology [8, 9], on transcription changes and regulation.

Since RNA sequencing’s introduction as an essential molecular biology tool, many technological iterations have occurred. From the initial iteration of sequencing by synthesis of cDNA, through improved technologies such as 454 pyrosequencing [10], and eventually the Illumina sequencing platform [11], a number of limitations have been identified. In particular, the need to fragment longer RNA polynucleotides and reverse transcribe the RNA and PCR amplify the cDNA before sequencing makes identification of RNA modifications [12], and variations in RNA structure and RNA splicing [13, 14] challenging [15, 16, 17].

Recent technological developments show promise in addressing many of the limitations associated with RNA sequencing. Specifically, PacBio and Oxford Nanopore Technologies (ONT) sequencing platforms have made long read—whole molecule—polynucleotide sequencing possible [18, 19, 20, 21, 22]. One such promising development is the Nanopore platform’s capability of sequencing native RNA polynucleotides in their whole form, without the need for replacement by cDNA and subsequent amplification [23, 24, 25]. Although Nanopore sequencing is capable of sequencing cDNA and cDNA amplicons [25], the sequencing of the native RNA strand (direct RNA-seq) allows the resolution of modified RNA nucleotides to provide context to the epitranscriptome [19, 26], while reducing the library preparation and analysis complexity [27, 28].

Over 100 unique RNA modifications have been described [29], including adenosine-to-inosine (A-to-I) edits, pseudouridylation, and methylation on multiple sites on the nucleotide base [30, 31, 32, 33, 34, 35]. Non-coding RNAs (ncRNAs) constitute frequent targets of these modifications with emerging significance for human health and disease. For example, higher A-to-I editing ratios in SINE RNAs have been linked to reduced severity in some viral infections such as by SARS-CoV-2 [36], and reduced A-to-I editing ratios have been linked to multiple sclerosis [37]. Additionally, m6A modifications are thought to encourage circular RNA formation by back splicing [38], and inosines in tRNAs allow for wobble base pairing for redundant codon recognition, dysfunction of which may cause intellectual disability [39]. Direct RNA-seq has exhibited the ability to detect these RNA modifications in recent research studies [28, 40, 41] by classifying perturbations in the ionic trace produced by Nanopore sequencing.

Despite the promise of Nanopore sequencing to deconvolute RNA modifications, the standard direct RNA-seq approach of using poly(A) selection during Nanopore library preparations, limits the capture of many ncRNAs that constitute the vast majority of known and conserved editing substrates [23, 32, 42, 43, 44]. Some of the substrates that may be omitted include among others tRNAs, snoRNAs, snRNAs, scRNAs, and other cellular non-poly(A) RNAs as well as viral RNAs. While targeted sequencing of ncRNAs has been employed in some instances, such as to sequence 7sk RNA [45] or rRNA [46], targeted sequencing still requires custom adaptors to the 3′ end of the sequence and is largely performed on one or a few transcripts at a time. Here, we present an approach, called NERD-seq, that expands the ncRNA representation in Nanopore direct RNA-seq to include multiple additional classes of ncRNAs genome-wide, while maintaining at the same time the ability to sequence high library complexity mRNA transcriptomes.

## Results

### Development of a direct RNA-seq library construction protocol (NERD-seq) to bypass the limitations posed by the standard direct RNA-seq approach

**Fig. 1:**
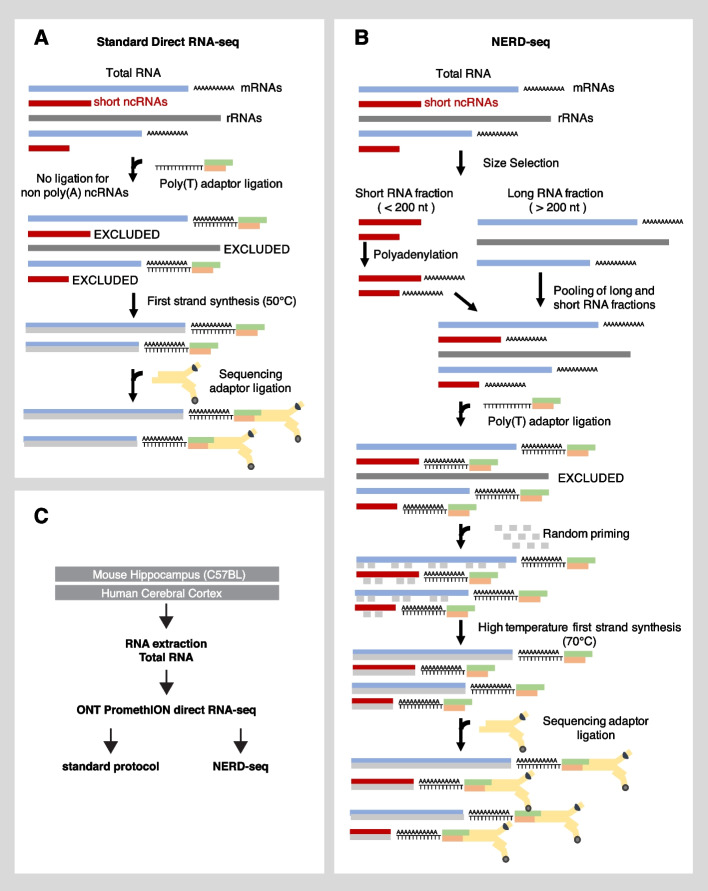
Description of the NERD-seq approach. **A** Illustration of the standard nanopore direct RNA-seq approach and the types of RNAs it can theoretically detect. ncRNA: non-coding RNA; rRNA: ribosomal RNA; mRNA: messenger RNA. **B** Illustration of the NERD-seq approach and the additional types of RNAs (short non poly(A) ncRNAs) it can theoretically detect. **C** Experimental design of the current study

Standard direct RNA-sequencing (RNA-seq) relies on poly(A) selection by using a poly(T)-tethering adaptor that base-pairs canonically to a motor protein linked adaptor, facilitating the movement of the polynucleotide string through the protein pore (Fig. 1A). While standard direct RNA-seq provides a method to sequence the poly(A) transcriptome, which predominantly represents post-transcriptionally modified mRNAs [23], it limits the detection of many short ncRNAs, that do not have a poly(A) tail. This is despite many of them being a primary target of RNA modifications that this new sequencing method aims to help detecting [47]. As mentioned below, the standard method may also limit the detection of ncRNAs that are highly structured and cannot get linearized in the temperature used during the reverse transcription step (50 °C).

To improve the representation of the above ncRNAs, while maintaining the ability to sequence the poly(A) transcriptome, we developed the Non-coding Enriched RNA Direct sequencing approach called NERD-seq. In this modified approach (summarized in Fig. 1B), we first separate PNK-treated total RNA into two fractions (long and short RNA fraction) using a column-based size enrichment approach, which we have used in the past for approaches that enable sequencing of also shorter ncRNAs (approx. < 200 nt) with Illumina sequencing (short-RNA-seq) [48, 49, 50]. This column-based approach enables for the separation of short transcripts from rRNA, so that they can be used in the subsequent polyadenylation step [51]. To the short fraction, we added a polyadenine tail using a poly(A) polymerase to allow in subsequent steps the short ncRNAs to form canonical base-pairing to the poly(T) sequencing adaptor and, thus, facilitate its ligation. Additionally, this poly(A) addition helps achieve higher accuracy in Nanopore sequencing of short RNAs because the beginning of the sequence usually requires some voltage adjustment for accurate basecalling and, proportionally, the error rate becomes higher for short reads [52, 53]. At the same time, full-length rRNAs that remain at the long RNA fraction, and are usually not desirable due to their extremely high read numbers that decrease yield for the other RNA classes, are not subjected to the polyadenylation, enabling their exclusion at later protocol steps. After deactivating the poly(A) polymerase, the two fractions (short and long) were pooled again together. Thus, the new sample contains all the initial short and long RNAs with a poly-A plus the fraction of the short ones that have been poly-adenylated by us.

An additional problem that arises in both Illumina and Nanopore sequencing protocols is the ability of highly structured RNA regions to hinder the sequencing process in various ways. In the case of Nanopore sequencing, highly structured RNA regions may prevent the pulling of the RNA molecule through the pore [25]. Although the direct RNA-seq protocol does not sequence cDNA, a first-strand cDNA synthesis step through a reverse transcriptase is recommended to stabilize the native RNA strand and resolve highly structured RNA regions commonly observed in SINE RNAs, snoRNAs and other ncRNAs (the cDNA strand is not sequenced) [54, 55, 56]. However, the direct RNA-seq protocol utilizes reverse transcriptases with a temperature optimum between 45 and 50 °C. At this temperature range, some highly structured RNA regions remain unresolved, resulting in reverse transcriptase pauses and a subsequent decrease in the length of sequenced transcripts [57]. To this end, we modified the complementary strand synthesis in two ways: Firstly, we reverse transcribed using the GspSSD2.0 DNA polymerase (Optigene, GSPSSD2-002HC). GspSSD2.0 is capable of reverse transcription at a temperature as high as 70 °C, which facilitates RNA unfolding. Moreover, to prevent RNA degradation at this temperature, we added random primers and initially allowed first-strand synthesis by GspSSD2.0 for 10 min at 50 °C to “coat” RNAs with protective cDNA strand fragments in multiple locations along their whole length. Since GspSSD2.0 has a strong strand displacement activity, all these short first-strand fragments are subsequently displaced by the reverse transcribed cDNA strand that is initiated by the poly(T) adaptor.

In order to explore further the strengths and limitations of our approach, in addition to GspSSD2.0, we have also tested other enzymes with similar strand displacement and high-temperature activities for their potential use with NERD-seq. These include the Omniamp and Lavalamp enzymes from Lucigen. The Omniamp enzyme performed well for our proposed methodology but it is not anymore commercially available. Results from screening with Omniamp can be found in Figs. S1–4. On the other hand, Lavalamp was found to have overall a reduced throughput of successfully based called reads (Additional file 1: Fig. S5A–B). Given our aim to be able to identify highly structured ncRNAs, such as snoRNAs, snoRNA class enrichment was initially used as a measure of effectiveness during screening for different enzymes and comparing different iterations of our approach (Additional file 1: Fig. S5C), revealing GspSSD2.0 as the best performing enzyme. Furthermore, in order to test whether we could possibly enrich short ncRNAs, mRNAs and long ncRNAs simultaneously, we also attempted to first deplete ribosomal RNA from the total RNA pool, polyadenylate the ribodepleted fraction and subsequently subject it to sequencing following the parameters of the NERD-seq protocol without size selection. Unfortunately, this strategy resulted in poorer yields (Additional file 1: Fig. S5D) in addition to poorer coverage (Additional file 1: Fig. S5E). Finally, we questioned whether the addition of A′ during the polyadenylation step in NERD-seq would generate an overall poly-A length bias compared to the standard approach; however, an analysis of the generated poly-A lengths compared to the corresponding transcript lengths of reads sequenced (Additional file 1: Fig. S5F) did not reveal a significant difference.

### NERD-seq enables the generation of reads with higher coverage for the non-coding genome, while still detecting mRNAs and poly(A) ncRNAs

To assess the potential of NERD-seq we selected total RNA from mouse hippocampus, a tissue that is very active at the transcriptome and epitranscriptome level [58, 59, 60], and performed both NERD-seq and standard direct RNA-seq on the same total RNA from this tissue (Fig. 1C). To our knowledge, there is only one study that has performed direct RNA-seq in neural tissues (whole brain) [61], and data from this study has been used as an external dataset for the validation of our findings (see Figs. S6–7). While in that study Sessegolo et al. used the first developed platform by ONT, called MinION, we used here our in-house ONT PromethION platform, which can produce approximately 10 times the yield of the MinION platform. This enabled for the first time, a more in-depth Nanopore sequencing in a neural mouse tissue, and the first direct RNA-seq in the hippocampus.

**Fig. 2:**
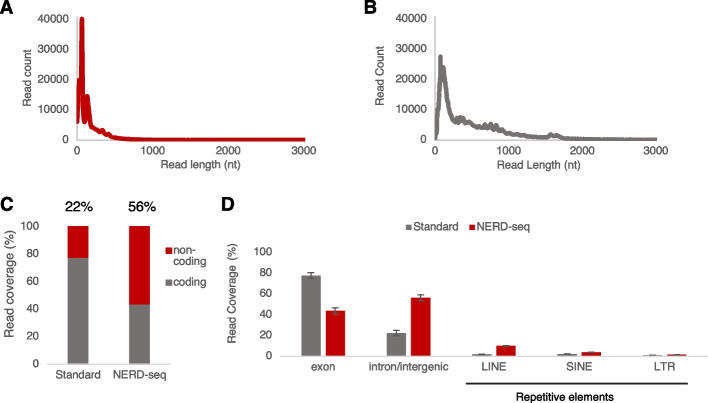
NERD-seq enables the generation of reads with higher coverage for the non-coding genome. **A**–**B** Comparison of the mapped read length distributions between the standard direct RNA-seq (**A**) and the NERD-seq approach (**B**). Both sequencing types were aligned to the mm10 genome using the minimap2 splice-aware mapping. **C** Percentage of reads aligning to the coding (exons) and non-coding portion of the genome in the standard and NERD-seq approach. Percentages were calculated as a proportion of total aligned reads. **D** Coverage (percentage) across various coding and non-coding genomic elements for standard and NERD-seq reads. Repetitive elements shown include reads mapping to LINE and SINE elements and long terminal repeats (LTR). Error bars depict standard deviation (*n* = 2)

Figure 2 shows the comparison between our NERD-seq approach and the standard direct RNA-seq approach with regards to read length metrics, confirming the inclusion of shorter RNAs through a reduction of the N50 of the NERD-seq approach to 354 nt compared to 1020 nt of the standard approach (Fig. 2A). These results demonstrate the efficient enrichment of shorter non-poly(A) RNAs by the NERD-seq protocol, thus enabling their analysis.

We then questioned how NERD-seq reads align to some of the most prevalent coding and non-coding genome elements. To this end, we calculated the percentage of the sequenced reads overlapping (> 80% of their length) to coding genes (known exons in mm10) or to regions outside of them (introns, intergenic regions). As shown in Fig. 2C, standard direct-RNA-seq reads predominantly (78% of the total reads) represent coding regions and particularly known exons, confirming that the standard approach is primarily tailored towards detecting and studying mRNAs and protein-coding genes. In contrast, this percentage falls to 44% in the case of NERD-seq, with the portion of reads coming from non-coding regions climbing from 22 to 56%. Finally, LINE elements are among the major non-coding elements that are overrepresented in NERD-seq compared to the standard approach (Fig. 2D).

**Fig. 3:**
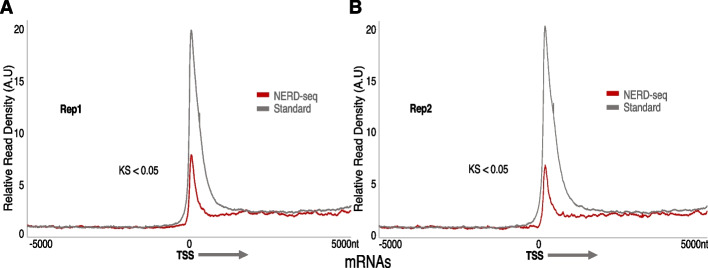
NERD-seq can reliably detect mRNAs. **A**–**B** Relative read density distribution around the Transcription Start Site (TSS) of known genes for standard and NERD-seq (representing replicate #1 and #2 in A and B, respectively). A metagene model has been constructed by aligning all known genes at their Transcription Start Site (as estimated by the use of Eponine at mm10 [110]) on the same strand. Distances at *X*-axis correspond to absolute distance (in nucleotides; nt) 500nt upstream (left) and downstream (right) from TSS. The arrow next to TSS depicts the direction of transcription. Read density is calculated by dividing the number of reads aligning to each position, divided to the total number of reads and elements (genes) that construct the metagene. KS: Kolmogorov–Smirnov test (KS) < 0.05 for the comparison between the two distributions. Both sequencing types were aligned using minimap2 with splice-aware mapping enabled

Despite the increase of the percentage of reads representing introns and intergenic regions in NERD-seq, reads originating from exons still constitute 44% of overall reads, suggesting that NERD-seq remains efficient in detecting mRNAs. To test this, we constructed metagene models of all known genes and compared the relative read density around their transcription site (i.e. read numbers normalized to the total number of sample reads and elements that constitute the metagene). The generation of distribution plots of relative read densities allows comparisons of read coverage among different samples for the same set of genomic elements that construct the metagene model. As shown in Fig. 3A, NERD-seq has been able to detect mRNAs as documented by the peak in the read distribution directly downstream of the Transcription Start Site (TSS). Consistent with the higher enrichment in mRNAs in standard direct RNA-seq vs. NERD-seq mentioned above, the relative height of the peak of the distribution at TSS is higher in the standard than in NERD-seq (Kolmogorov–Smirnov test (KS test) < 0.05).

Next, we asked whether NERD-seq can detect also ncRNAs that are known to be polyadenylated. To test this, we checked for the expression of ncRNAs generated by one of the most frequent mouse SINE elements, the B2 element, which has been described to be polyadenylated [62, 63]. As shown in Additional file 1: Fig. S8, NERD-seq can still detect full-length B2 RNAs.

We then sequenced a different tissue (cerebral cortex) from a different organism (human) and found that the mRNA read distribution is similarly replicated in pooled human cerebral cortex RNA (Additional file 1: Fig. S9–A) as well as another polyadenylated ncRNA orthologous to B2 RNA in humans: Alu SINE RNAs. In this case, Alu RNAs appear to be even more enriched in NERD-seq datasets than standard ones (Additional file 1: Fig. S9–B).

Since NERD-seq can capture mRNA reads, albeit at a lower coverage than the standard approach, we then questioned whether it could be used—like the standard approach—to generate information from mRNAs such as their splicing patterns. To check this, we spiked in the RNA sequins mix B [64] into both the standard and NERD-seq samples and used the Anaquin software analysis package [64] for sequins analysis with minimap2 mapped reads against a custom mm10 mouse genome with the sequins pseudochromosomes included. The counting outputs for both the NERD-seq approach and the standard approach suggested that the expected and observed abundances were similar between the standard and NERD-seq (Additional file 1: Fig. S10). Furthermore, using the IGV viewer for sequin gene R2_38, the standard, and NERD-seq approaches produce almost identical splicing patterns in their sashimi plots (Additional file 1: Fig. S10). In addition, when we assess all mRNAs without the minimap 2 splicing parameter, the profile between the no splice mapping and splice-aware mapping for NERD-seq are slightly different, confirming the ability of NERD-seq to detect mRNA splicing (Additional file 1: Fig. S11).

Overall, our findings show that NERD-seq can efficiently detect poly(A) transcripts and their isoforms detected by the standard protocol.

### NERD-seq can efficiently detect various classes of short non-poly(A) ncRNAs in contrast to the standard approach

We then questioned whether NERD-seq is able to also detect known classes of non-poly(A) ncRNAs, which was among our primary motivations for developing the NERD-seq method. To this end, we first generated the relative read density distribution plots around the TSS of the following four classes of ncRNAs: snoRNAs, snRNAs, scRNAs, and srpRNAs. As mentioned above, for validating our results, in order to exclude any lab-specific technical systematic errors, in addition to the data generated by us through the standard approach for the same RNA pool, we have also employed external direct RNA-seq data generated from the same organ (brain) and the same standard approach [61] for the comparison with our NERD-seq data (presented in Additional file 1: Fig. S6–S7).

**Fig. 4:**
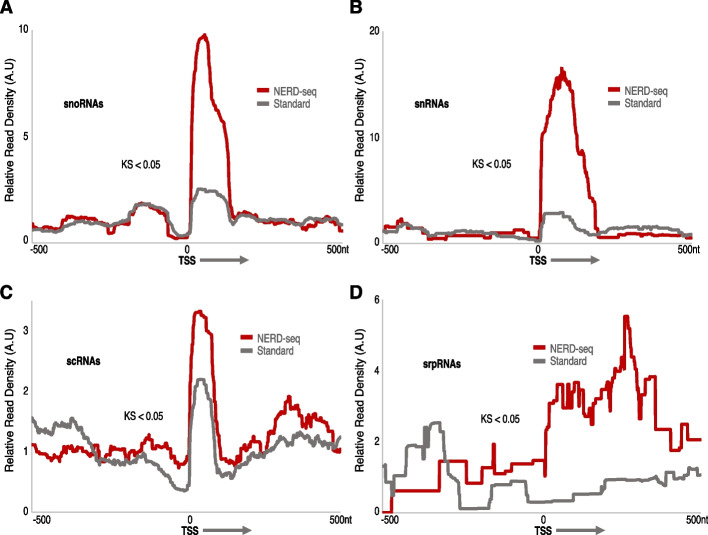
NERD-seq can detect snoRNAs, snRNAs, scRNAs, and srpRNAs. Metagene plots depicting relative read density around the transcription start site (TSS) for the following RNA classes: snoRNAs (**A**), snRNAs (**B**), scRNA (**C**), and srpRNAs (**D**). *X*, *Y*-axis, mapping, and KS-test as in Fig. 3. All plots depict 500nt upstream (− 500) and downstream [500] of TSS (0 nt)

As shown in Fig. 4, for three out of four of these classes, snoRNAs, snRNAs, and srpRNAs, the standard approach can hardly detect any of them, while it also significantly underperforms in the case of scRNAs (KS < 0.05) compared to NERD-seq. In contrast, NERD-seq produces robust distributions for all four classes (see also Additional file 1: Fig. S6–7 for a comparison with external data). We then compared our Nanopore data (NERD-seq and standard) to our previously published Illumina sequencing data on the same mouse hippocampus samples, that were optimized for the detection of short RNAs [48]. The differences between the metagene enrichment between NERD-seq and the standard approach exhibit a similar signature as the enrichment between the short RNA optimized and standard long RNA Illumina libraries (Fig. S12–15). Overall, the NERD-seq metagene plots exhibit replicable signatures between replicates (Fig. S12–15 A and B panels) in addition to exhibiting similar enrichment for snoRNA, snRNA, scRNA, and srpRNA classes between mouse samples and human cerebral cortex RNA data (Additional file 1: Fig. S16).

**Fig. 5:**
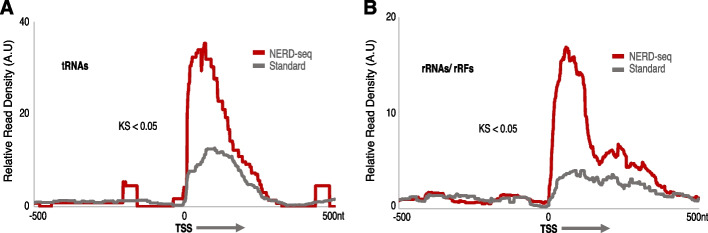
NERD-seq can detect tRNAs and small RNAs mapping to rDNA. Metagene plots depicting relative read density around the transcription start site (TSS) (mm10) for the following RNA classes: tRNAs (**A**) and rRNAs (**B**). *X*, *Y*-axis, mapping, and KS-test as in Fig. 3. All plots depict 500 nt upstream (− 500) and downstream (500) of TSS (0 nt)

Then, we asked whether NERD-seq can detect another important class of non-poly(A) ncRNAs: tRNAs, which are known and conserved RNA modification substrates. As shown in Fig. 5A, NERD-seq produces a robust distribution around the TSS of these RNAs (see also Additional file 1: Fig. S7 for a comparison with external data). Interestingly, the standard approach can still detect some of them, though almost threefold less than NERD-seq. Although tRNAs are known to be non-polyadenylated, it appears that they can still be detected by a poly(A)-selecting approach, such as the standard direct RNA-seq, presumably when they are marked with poly(A)s during degradation [65]. When we compare this with Illumina data and the human cerebral cortex data, a similar profile in the metagene is produced (Additional file 1: Fig. S17–18). As discussed below, this finding denotes the importance of an RNA-seq approach that can detect the non-poly(A) RNAs, such as tRNAs, as those may represent an entirely different biological context compared to those marked for degradation.

Finally, we examined the level of rRNAs in our data. As in the case of tRNAs, rRNAs are also marked with poly(A) tails for degradation, so even the standard approach is expected to detect some long rRNAs as shown in Fig. 5B, which also applies to NERD-seq. Interestingly, in addition to the background rRNA detected in the standard approach, NERD-seq distribution around the rRNA TSS depicts a strong peak approx. 120–140 nt wide, suggesting the capture of 5S rRNA, and large and short subunit derived short rRNA fragments generated from this position that are missed by the standard approach (see Additional file 1: Fig. S3 for a comparison with external data). Similar enrichment results were produced when testing the Illumina small RNA library and long RNA library, as well as the sequencing experiments on the human cerebral cortex dataset (Fig. S18–19) Such rRNA fragments called rRFs, have been described as being capable of modulating rRNA transcription and function [66]. As well, these fragments are important to cell survival and proliferation [67] in a sex, tissue, and population-specific manner [68].

Because the metagene plots produced in Fig. 5 and Additional file 1: Fig S12–15, 17, 19 exhibited enrichment in short RNA profiles, including also small RNAs which are shorter than 50nt, we produced a small RNA distribution plot for RNAs mapping to large and small subunit rRNAs, tRNAs, miscRNA, and microRNA genes (< 50 nt in size). Indeed, between the two replicates of NERD-seq in hippocampus mouse tissue and the two replicates of the standard approach in the same tissue, we see enrichment of small and large subunit rRNAs as well as miRNA precursor reads in NERD-seq datasets (Additional file 1: Fig. S13). Interestingly, while both RNA classes are also expressed in the same range in standard, the NERD-seq profiles in the small RNA QC plot appear to be better replicated than in the standard approach (Additional file 1: Fig. S20), suggesting NERD-seq is indeed better suited to capture shorter fragments and that many of the reads produced, which map to RNAs like tRNAs and small and large subunit rRNAs may in fact be rRF and tRF RNAs.

Throughout the study we have been able to replicate the above results in the same tissue (Fig. S12–15, 17, 19–20), and a different tissue and organism (Fig. S16 and 18). Altogether, these results show that NERD-seq can efficiently expand direct RNA-seq capabilities to detect multiple classes of short non-poly(A) ncRNAs that may be missed or sequenced in inadequate numbers by standard RNA-seq.

### NERD-seq allows for the detection of LINE 1-produced ncRNAs

**Fig. 6:**
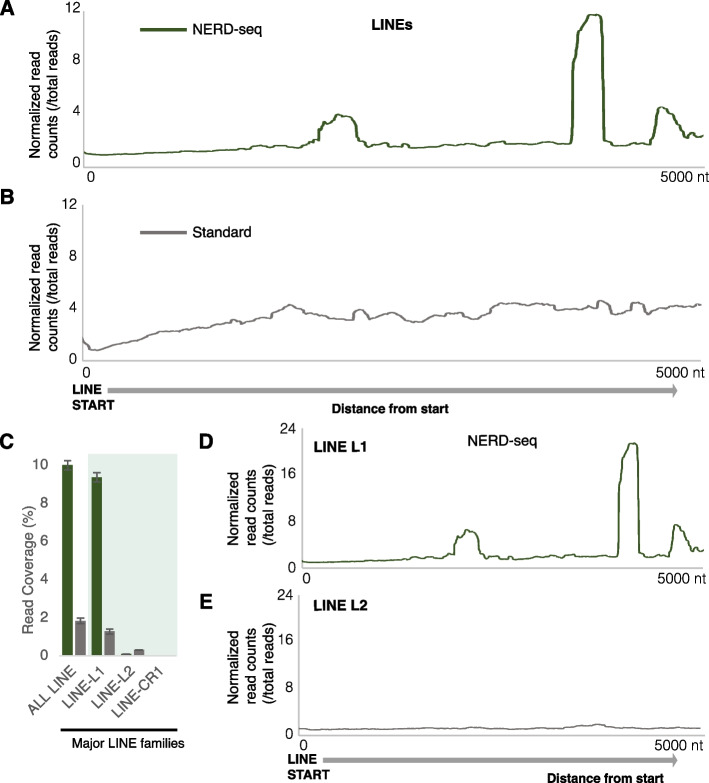
NERD-seq detects LINE L1-associated ncRNAs. **A** Normalized counts of reads from NERD-seq mapped across the first 5000 nt of a metagene constructed by all known LINE elements (repeat masker UCSC mm10). The metagene model has been constructed by aligning all known LINEs at their start site (base #1 in each element’s DNA sequence). Distances at *X*-axis correspond to absolute distance (in nucleotides; nt) 5000 nt downstream from the start. The arrow below the graph corresponds to the sense direction of the elements. Normalized read counts per position are calculated by dividing the number of reads aligning to each position to the total number of reads. **B** Same as in **A** but for standard direct RNA-seq reads. **C** Coverage (percentage) across all LINEs and across the three LINE subfamilies for NERD-seq reads. The three LINE subfamilies with the most aligned reads have been selected and are depicted here. Error bars depict standard deviation (*n* = 2 replicates). **D** Per million reads normalized counts of reads from NERD-seq mapped across the first 5000 nt of a metagene constructed as in **A** but by all known LINE L1 family elements. **E** Per million reads normalized counts of reads from NERD-seq mapped across the first 5000nt of a metagene constructed as in **A** but by all known LINE L2 family elements

As shown in Fig. 2C, reads mapping to LINE elements are overrepresented in NERD-seq. ncRNAs from LINEs have been described as important for preventing neurodegeneration with its interaction with homeoprotein b in dopaminergic neurons [69]. Also, they function to aid SINE RNAs in retrotransposition [70, 71]. LINEs span genomic regions around 5 KB or more. We aimed to identify hotspots across the LINE elements producing the RNAs detected by NERD-seq. To this end, we mapped reads generated by both the NERD-seq and standard approach across the LINE metagene, which was constructed by aligning all known LINE elements in mm10 at their transcription start site (Fig. 6A and B, respectively). As shown in these figures, consistent with our findings in Fig. 2C, reads mapping to LINE elements in NERD-seq data are enriched compared to the standard library. We then attempted to pinpoint the exact LINE elements producing these ncRNAs. To this end, we compared the read coverage of NERD-seq reads across all the major LINE elements listed in the UCSC mm10 repeat masker annotation track for the top-ranking identity based on read coverage. As shown in Fig. 6C, NERD-seq read coverage of LINE elements comes predominantly from the L1 family, which far outweighs the following two families. This is confirmed by plotting NERD-seq reads at the respective L1 metagene (Fig. 6D) and comparing this plot with that of other LINE families, such as L2 (Fig. 6E). Because the metagene plots suggest full-length L1 reads are not being captured in either library but rather shorter sequences within L1 elements, we determined which L1 reads were most commonly mapped and found that L1Md_T (mm10—chr13: 9,832,020–9,838,665) is most enriched in the NERD-seq library. When we examined this genomic region, a polyadenylated genomic sequence that closely matches a portion of the 28S rRNA genomic sequence was found to be enriched (Additional file 1: Fig. S21) and using a pairwise alignment algorithm (Emboss Water) between the consensus sequence extracted from IGV viewer and the mouse rDNA sequence (NCBI Refseq: BK000964.3), we found a 99.2% identity match (Additional file 1: Fig. S22). Furthermore, when we used the UCSC BLAT alignment tool on the identified sequences within the L1Md_T element, we found that the reads map closely to multiple regions identified as the recently described LSU-rRNA_HSA SINE repeats which are ancestrally derived from the 28S rRNA and have retained much of the sequence’s fidelity (Additional file 2: Table S1) [72]. Once again, the enrichment of these elements is similarly exhibited by the comparison of the Illumina sequenced small RNA and long RNA libraries in Additional file 1: Fig. S16.

These results reveal the ability of NERD-seq to detect ncRNAs derived from within the LINE elements and how it may enable the extraction of more information from sequencing reads, not available through the standard approach.

#### NERD-seq expands the study of epitranscriptomic signatures to more ncRNA classes

**Fig. 7:**
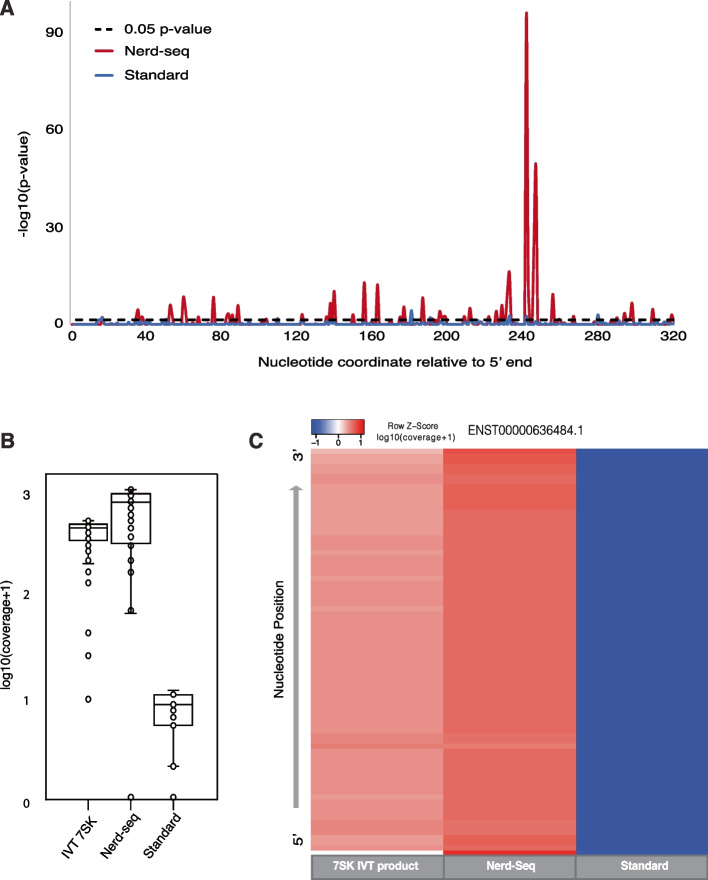
NERD-seq detects RNA modifications in 7SK RNA. **A** Line plot depicting the inverse log of the Student’s *t*-test *p*-value of all kmers between NERD- seq and IVT (red), and Standard and IVT (blue). The black dotted line denotes the 0.05 *p*-value cutoff. **B** Box and whisker plot depicting the log(coverage + 1) of each kmer from 7SK. **C** Heatmap depicting the coverage of each kmer by nucleotide position in 7SK. Values are normalized by row and taken as a function of log(coverage + 1)

To assess whether use of data produced through the NERD-seq protocol is capable of detecting RNA modifications, we in vitro transcribed (IVT) the human rn7SK (Esembl: ENSG00000283293) gene from a synthetic construct and used it as a comparative dataset to the total RNA sequenced with both the standard and NERD-seq protocol from a [73] 5-donor pooled source of RNA from human cerebral cortex. We then used XPore tool to parse kmers by transcript and determined which kmers were producing significantly different electric current distributions (Fig. 7A). Doing so, we identified highly significant differences in the HP3 stem-loop region which was also found by Leger et al. [45] by comparing (GGTCC position 242, *pval* = 5.10E − 97; GTCCA position 243, *pval* = 2.00E − 12; CATTT position 246, *pval* = 8.23E − 27; ATTTG position 247, *pval* = 7.96E − 50). As well, multiple kmers that produced significant differences in m6A mapping experiments between an unmodified sample and a Mettl3 knockdown (reported in [45]) were reproduced, including A56 (GUUGA, *pval* = 6.97E − 04), A77 (GCUAG, *pval* = 3.44E − 09), A151 (GACG**A**, *pval* = 8.41E − 04), A162 (G**A**UAG, *pval* = 4.75E − 13), A186/187 (CAAGG, *pval* = 5.16E − 09) A200 (AGT**A**G, *pval* = 3.58E − 03), A230/231 (AACAA/CAAGC/AAGCU, *pval* = 1.06E − 06/4.89E − 03/1.06E − 11), and A245 (GTCCA/CATTT/ATTTG, *pval* = 2.00E − 12/8.23E − 27/7.96E − 50) [45].

When we compared the 7SK IVT reads to the standard protocol, we were unable to capture enough 7SK reads to reproduce modification sites (Fig. 7B–C). And, while Leger et al. [45] was able to sequence 7SK from cell line experiments, it required a targeted direct RNA sequencing approach with a custom 7SK 3′ end-specific adaptor, whereas using NERD-seq we were able to capture these reads from 7SK, in addition to various snoRNAs, snRNAs, scRNAs, srpRNAs, tRNAs, shorter rRNAs, B2 RNAs and mRNAs without the need for a special adapter for each one of them (Figs. 3, 4, and 5).

Moreover, when we view the rn7SK genomic region in the hg38 genome, we have been able to confirm a strong T/C mismatch in the pseudouridine site known to occur in Uracil 250 (Additional file 1: Fig. S23) which has been previously reported for its importance in snRNP formation [74]. T/C base calling mismatches (genomic T) have been shown to correlate with pseudouridine sites in direct RNA sequencing datasets [46].

Furthermore, the RNU5F-1 transcript has been shown to harbor pseudouridine sites in nucleotides U43, U46, and U53 [75], all of which exhibit genomic T T/C mismatches (Additional file 1: Fig. S23). Interestingly, in nucleotide 64, a genomic A features a strong A/G mismatch signal which has also been shown to be indicative of inosine sites [23]. Of course, additional, unexplained mismatch sites are produced and require characterization, such as C/T mismatches (genomic C) in C32 and C36, both of which could be APOBEC substrates, producing a cytosine to uracil conversion [76]. Lastly, additional T/C (genomic T) mismatches are present in U40–42, suggesting previously unexplained pseudouridine sites, or possibly a base calling error because they reside in a homopolymer uracil region, known previously to basecall less accurately [77]. Similar to 7SK RNA, the standard approach was unable to capture enough RNUF5-1 to enable profiling of the transcripts’ epitranscriptomic signatures.

### NERD-seq exhibits similar enrichment in SQK_RNA004 direct RNA sequencing libraries

The initially released direct RNA sequencing products from Oxford Nanopore Technologies performed sequencing using the “R9” flow cell whereby direct RNA sequencing libraries are prepared using the SQK_RNA001 library kit, or like in this study, using the SQK_RNA002 library kit. A follow-up to these technologies is the RNA-specific flow cell released by ONT in combination with the SQK_RNA004 library kit. To ensure NERD-seq is a protocol that can be used well into the future, we assessed also the enrichment of non-coding RNAs in a library prepared using the SQK_RNA004 library kit with the RNA-specific flow cell. As exhibited in Additional file 1: Fig. S24, and similarly to what we demonstrate in the SQK_RNA002 library preparations with the R9 flow cells, the NERD-seq preparations enrich snoRNAs, snRNAs, scRNAs, and srpRNAs.

## Discussion

Expanding our understanding of the epitranscriptome and its constituting RNA modifications, and why and where RNA edits occur, is expected to have a profound influence on our understanding of how cells use RNA in different signaling and functional contexts [78, 79], and how cells recognize internally transcribed RNAs compared to dysfunctional RNAs and RNA-related threats. The Nanopore platform and direct RNA-seq have opened new avenues for such studies. However, few library approaches have been developed to leverage the potential of Nanopore’s technology to detect ncRNA classes. Of the few methodologies developed, they largely focus on a single RNA class or transcript, including the targeted RNA sequencing approach with 3′ end-matching adaptors [45]. Here, we present an approach that enables the simultaneous enrichment of multiple classes of ncRNAs which constitute important RNA modification targets. NERD-seq can not only successfully detect mRNAs and polyadenylated ncRNAs detected by the standard approach, but it also expands the detection of ncRNAs to all major classes of short non-polyadenylated ncRNAs. At the expense of reducing coverage of protein-coding RNAs for only 34% (a decrease of 78% of overall reads to 44%), NERD-seq allows the successful detection of snoRNAs, snRNAs, scRNAs, srpRNAs, tRNAs, and other ncRNAs. Moreover, while not amplifying full-length rRNAs, which is often undesirable, it can still detect rRFs from small and large subunit rRNAs that have recently received attention for their critical roles in various biological contexts such as cell proliferation and survival [67] and rRNA transcription modulation [66]. As well, NERD-seq demonstrably improves the capture of a relatively understudied RNAs such as the SINE RNA called LSU-rRNA_HSA, providing a promising means to assess these transcripts.

For our comparison between NERD-seq and the standard protocol, we initially chose mouse hippocampal tissue due to its rich epitranscriptome variability throughout neurodevelopment and upon conditional treatment [58, 59, 60]. Hippocampi exhibit high transcriptional activity and genome-environment interactions, while also being responsive to environmental and physiological changes [80, 81, 82, 83]. Additionally, it is a tissue that has received considerable attention for its role in memory formation and learning, and its early dysfunction in disorders such as Alzheimer’s and Parkinson’s disease and during cancer treatment [48, 49, 84, 85, 86, 87, 88, 89]. Moreover, we were able to demonstrate the replicability of our methodology in human cerebral cortex tissue.

Our approach has enabled for the first time, in-depth Nanopore PromethION sequencing in a neural mouse tissue, and the first direct RNA-seq in the hippocampus with both the standard and our new NERD-seq approach. All classes that are detected by NERD-seq have been shown to bear critical roles in neural cells [90, 91], and, thus, by expanding capabilities for studying their epitranscriptomes, NERD-seq will help further elucidate the mechanisms underlying neural RNA regulation. For example, snoRNAs have been shown to be key players in the RNA modification machinery [92] but, until now, the standard approach does not allow to test for the impact of such changes on snoRNAs themselves in terms of potential self-regulatory loops. Moreover, marking of ncRNAs with poly(A) for degradation makes them the only ncRNAs in their class to be detected by the standard sequencing methods, adding a significant confounding factor, as any RNA modifications detected may be only connected with their degradation process and not with their other functions. NERD-seq can now identify those otherwise undetectable ncRNAs and increase the overall portfolio of RNAs.

Using RNA from the human cerebral cortex, we also demonstrated that NERD-seq is able to produce high enough sequencing coverage and resolution to detect epitranscriptomic signatures such as signatures in known m6A and pseudouridine sites in the 7SK RNA [45] and in known snRNA RNU5F-1 pseudouridine sites [75]. For both transcripts, the standard direct RNA-seq approach was unable to produce enough coverage for robust RNA modification detection. Thus, while the standard approach is still customized for mRNAs and thus may require lower sequencing coverage for resolving RNA modifications in mRNAs, the study of RNA modifications in other important RNA species that is improved by the NERD-seq protocol offers a more comprehensive picture of the epitranscriptome. Indeed, there is high potential to expand the study of RNA modifications in mammalian transcriptomes using NERD-seq. For instance, in the modomics RNA modifications database, 20 transcripts are described in the mouse transcriptome for snoRNAs, snRNAs, and tRNAs. This is compared to 103 entries in *Saccharomyces cerevisiae* [93], demonstrating a need for high throughput assessment of ncRNA modification signatures in mammalian transcriptomes. Because of the development of ONT technologies, projects like directRMDB (direct RNA modification database) have been developed, annotating many RNA modification sites discovered with direct RNA-sequencing strategies [94]. However, the RNA modifications deposited are largely focused to mRNAs, whereas utilizing sequencing strategies like NERD-seq, paired with robust comparative RNA samples, like what is used to detect RNA modifications with tools like Epinano, Xpore, and Nanocompore will be a useful addition to the Nanopore sequencing repertoire, allowing for the assessment of RNA modifications in multiple classes of ncRNAs simultaneously [45, 95, 96].

Nevertheless, this study leaves unanswered some important questions. For example, NERD-seq is able to detect more ncRNAs derived by LINEs compared to the standard approach, a finding that we have narrowed down to L1 elements; however, this finding is complicated by the large length of L1 elements making unclear the identity of these L1-associated RNAs and whether they are related with previous ncRNAs described in LINEs. In some instances, we have identified these reads as belonging to the LSU-rRNA_HSA SINE element in mice but we have not been able to exclude that other RNAs may also reside within these elements. It also remains unclear why NERD-seq is able to enrich inner-LINE elements. Potentially, the use of high-temperature reverse transcriptases like GSPSSD2.0 or omniamp enzymes—as used in this study—enable the resolution of highly structured RNAs produced from within these elements that had been missed from other approaches. Coincidentally, the Illumina data we present shows similar enrichment patterns between long and short fractions for the LSU-rRNA_HSA but are unable to resolve the full-length transcript, indicating both that there may be shorter transcripts arising from these elements and that the full length and possibly more structured RNA species cannot be resolved by lower temperature reverse transcriptases. Other RNA candidates which could account for some of the read enrichment in the LINE annotations elsewhere include U6 RNA, which has been shown to recruit RtcB to ligate U6 RNAs to L1 RNAs, resulting in U6 pseudogene formation. Also, the SVA SINE element is known to recruit the L1 machinery for non-autonomous retrotransposition and has been shown to cause neurodevelopmental disorders due to insertional mutagenesis [97]. As such, NERD-seq is a promising approach to the study of LINE L1-derived ncRNAs and further attention to these datasets may result in novel discoveries of ncRNA transcription dynamics from these genomic elements.

While we are able to capture reads known to have high structure complexity, likely because of a high-temperature first strand cDNA synthesis to resolve complex structures into more linear structures, the reliance on polyadenylation enzymes presents a possible source of read bias. For instance, stem-loop structures, which are common in some ncRNAs, like riboswitches [98] on their 3’ ends are known to inhibit the polyadenylation reaction [99].

A limitation of standard Nanopore direct RNA sequencing, which is retained in NERD-seq, is that it cannot capture non-polyadenylated, long RNAs. And, while techniques are available to capture these RNAs one to a few molecules at a time [100], or in the case of nascent RNAs using chromatin isolation [101], the ability to capture simultaneously all reads with an existing current Nanopore sequencing technique is still not present. However, as the methodology of direct RNA sequencing matures and additional library approaches, such as NERD-seq are developed, there are expected improvements in transcriptome capture using the Nanopore platform, similar to the development of total RNA sequencing approaches available with Illumina chemistry [102]. Moreover, the MinKNOW sequencing software ONT uses to operate its instruments and package its basecaller, has been shown to exclude shorter RNAs, classifying them as adaptor sequences, and by adjusting the filtering parameters, it is possible to produce more reads in the final output [103]. How adjusting the MinKNOW filtering on a NERD-seq dataset would adjust its final output is unclear. However, it is apparent that NERD-seq libraries do reliably capture reads shorter than 50 nt, indicating the library may be improving the lower-end detection limit, potentially because of the polyadenylation reaction conducted on RNA molecules in the short RNA fraction. Furthermore, ONT has very recently developed an RNA-specific motor protein with itsRNA004 chemistry. It has been reported that short read capture and read throughput are improved in RNA004 [104] and as shown in Additional file 1: Fig. S24 we have been able to replicate our results also by using the new RNA chemistry RNA004 flowcells and observing through NERD-seq the same benefits as with the R9 flowcells. Because NERD-seq improves the capture of short RNAs and its advantages remain as this sequencing technology develops, it is a methodology with the potential for long-time utility. To this end, NERD-seq is a promising development to address many of the limitations of standard direct RNA-seq due to its ability to capture multiple classes of short ncRNAs and highly structured RNAs, hitherto only achievable one transcript at a time with custom adaptor ligation. Thus, based on our findings, NERD-seq presents a simple but powerful approach for transcriptome and epitranscriptome analysis that expands our ability to exploit the potential of Nanopore sequencing technology.

## Conclusions

The development of direct RNA sequencing has generated vast interest due to its potential to unveil epitranscriptomic signatures in RNA molecules. With the standard preparation of direct RNA sequencing libraries, many ncRNAs that are important regulatory elements and are substrates for epitranscriptomic markers like RNA modifications are omitted. By advancing the direct RNA sequencing protocol for the simultaneous detection of various classes of ncRNAs alongside polyadenylated RNAs such as mRNAs, we demonstrate the potential of Oxford Nanopore sequencing’s direct RNA sequencing technology to quantify ncRNAs and to assess their epitranscriptomic signatures. The protocol herein, called non-coding enriched RNA direct sequencing (NERD-seq) advances the standard direct RNA sequencing library technique to enrich and capture multiple classes of ncRNAs simultaneously with mRNAs. We demonstrated the utility of NERD-seq by quantifying the relative enrichment of important ncRNA classes like snoRNA, snRNA, scRNAs, srpRNAs, and RNAs expressed from rRNA, tRNA, and L1 LINE element genes. As well, we assessed snRNA epitranscriptomic markers such as likely N6-methyladenosine and pseudouridine sites in 7SK RNA in addition to resolving known pseudouridylation sites in RNU5-F. Altogether, the NERD-seq methodology is a useful addition to the Oxford Nanopore sequencing library approach repertoire and will enable a more comprehensive transcriptome and epitranscriptome analysis by improving the study of many important ncRNAs alongside the standard analysis of mRNAs.

## Methods

### Hippocampal tissue RNA extraction

Mice were raised and had tissue extracted as described previously [48]. Left and right mouse hippocampus tissue were homogenized separately in 1.0 mL TRIzol reagent: 15-min incubation and subsequent grinding using a pestle until nothing but insoluble connective tissue remained. The homogenized mix was pipetted up and down and the solution was stored at – 80 °C. 0.5 mL of the homogenized mixture was phase separate by the addition of 100 µL of chloroform (Sigma, C2432) and mixed by inversion, incubated for 3 min, and centrifuged at 12,500 × g for 15 min at 4 °C. The top (aqueous) layer was transferred to a new tube and mixed with 250µL of isopropanol (Fisher, 67–63-0), followed by a 1-h incubation at – 20 °C and centrifugation at 12,500 × g for 10 min at 4 °C. The supernatant was removed and the pellet was washed and mixed with 0.5 mL of 75% ethanol, followed by a centrifugation at 7600 × g for 5 min at 4 °C. The supernatant was removed and the pellet was allowed to dry for 1 min before eluting in 30 µL of nuclease-free H_2_O. The eluted RNA was heated at 55 °C for 15 min, and subsequently incubated with 1 µL of DNaseI (NEB, M0303), 10 µL of 10 × DNaseI buffer (NEB, B0303), and 39 µL of nuclease-free H_2_O for 15 min at 37 °C. The RNA was further cleaned using the Zymo Research RNA clean and concentrator kit -25 (R1017) and combined in equal densities (left and right RNA). The RNA was stored at – 80 °C. Two biological replicates have been used throughout the study (mentioned simply as “replicates” across the text).

### Other RNA sources

Cerebral cortex total RNA from humans were sourced through Takara Biosciences (636,561). For libraries sequenced with the SQK_RNA004 kit as indicated, mouse hippocampus sourced from Takara Biosciences was used (636,663). Synthetic 7SK RNA was produced using the following custom g-block from IDT (T7 promoter is represented by lowercase letters at the 5′ end of the template): 5′ -taatacgactcactataGGATGTGAGGGCGATCTGGCTGCGACATCTGTCACCCCATTGATCGCCAGGGTTGATTCGGCTGATCTGGCTGGCTAGGCGGGTGTCCCCTTCCTCCCTCACCGCTCCATGTGCGTCCCTCCCGAAGCTGCGCGCTCGGTCGAAGAGGACGACCATCCCCGATAGAGGAGGACCGGTCTTCGGTCAAGGGTATACGAGTAGCTGCGCTCCCCTGCTAGAACCTCCAAACAAGCTCTCAAGGTCCATTTGTAGGAGAACGTAGGGTAGTCAAGCTTCCAAGACTCCAGACACATCCAAATGAGGCGCTGCATGTGGCAGTCTGCCTTTCTTTTACATATAATAAATAAATAAATCTTTAAAAAAAAA – 3′. The g-block was prepared for in vitro transcription as described previously [48]. Synthesized RNA was assessed for purity using the Agilent Bioanalyzer RNA pico assay and 1000 ng was subsequently polyadenylated as described below. Afterwards, the RNA was sequenced using the standard direct RNA sequencing approach described below.

### Direct RNA sequencing using Nanopore

For the standard approach, we used the ONT SQK_RNA002 kit and the direct RNA-seq protocol, listed at the Nanopore Community portal with 1.5 µg total RNA as starting material. Where indicated, we also used the SQK_RNA004 kit, following the same modifications for NERD-seq and following the manufacturer’s recommendations for the standard sequencing libraries. In both the standard and the NERD-seq protocol, ligations were performed for 15 min.

For NERD-seq, the standard protocol was further modified as follows: 1.5 µg total RNA was PNK digested using 50U PNK (NEB, M0201) and 10 µL 10X PNK buffer for 1 h at 37 °C, then directly separated into two fractions (short and long RNA) using the Invitrogen MirVana kit (AM1561) with a modified protocol as described before in our short RNA-seq approach for Illumina [48, 50, 105, 106]. Ninety microliters of the short RNA elution fraction was polyadenylated using 12 µL 10 × polyA polymerase buffer (NEB, B0276), 12 µL 10 mM ATP (NEB, B0756A), 6 µL polyA polymerase (NEB, M0276), and incubated at 37 °C for 30 min. The polyadenylated short fraction and the long fraction were combined, purified, and concentrated using the RNeasy MinElute kit (Qiagen, 74,204) to a final volume of 12 µL of RNA that was adaptor ligated using 4 µL NEBNext Quick ligation buffer (B6058), 0.66 µL RNA CS (Nanopore), 1.3 µL RTA adaptor (Nanopore), 2 µL 2,000,000U T4 DNA ligase (NEB, M0202). The reaction was incubated at room temperature for 15 min. The sample was subsequently reverse transcribed using 150U GspSSD2.0 DNA polymerase (Optigene, GSPSSD2-002HC), 10 μL 10 × buffer, 8 μL dNTP (NEB, N0447), 12 μL 50 mM MgSO4, 10 μL Betaine, 5 μL random primer mix (NEB, S1330), and 33.5 μL nuclease-free H_2_O. The mixture was incubated for 50 ℃, 10 min, and 70 ℃, 20 min. Samples were cleaned using 2.88 × Omega Mag-Bind® TotalPure NGS beads and eluted in 23 μL. The 23-μL library was ligated to the Nanopore RMX adaptor using 6 µL RMX adaptor (Nanopore), 8 µL NEBNext quick ligation buffer, and 3 µL 2,000,000U T4 DNA ligase and incubated for 10 min at room temperature. The adapted library was 1 × bead cleaned using the Omega Mag-Bind® TotalPure NGS beads, eluted in a Nanopore elution buffer. The library was loaded onto Nanopore PromethION according to the manufacturer’s instructions (flow cells were version 9.4.1).

For the NERD-seq version that employed reverse transcription by the OmniAmp polymerase, the NERD-seq protocol mentioned above was modified using 1.5 µL of the Lucigen OmniAmp polymerase (F831942-1), 10 µL 10 × OmniAmp buffer (Lucigen, F883707-1), 8 µL dNTP (NEB, N0447), 6 µL 100 mM MgSO4 (Lucigen, F98695-1), 10 µL Betaine (Lucigen, F881901-1), 5 µL random primer mix (NEB, S1330), and 39.5 µL RNase-free H_2_O. The mixture was incubated for 50 ℃, 10 min, and 70 ℃, 20 min.

### Read mapping

The RNA sequencing data was aligned using minimap2 [107] version 2.17 against the GRCm38/mm10 mouse genome or the GRCh38/hg38 human genome. Minimap2 was used with the options: -ax sr to optimize for short RNA reads in the case of ncRNAs and using the -ax splice -uf -k14 options to optimize for gene splicing to study mRNAs. Aligned sam files were converted to bam format using samtools version 1.7 [108]. Bam to bed conversion was performed using bedtools version v2.26.0 [109]. Intersections were performed using bedtools v2.26.0 with the intersect options: -s -wa -u -e -f 0.8.

### Metagene plots

Annotations for LINE RNAs, scRNAs, scRNAs, srpRNAs, rRNAs, Alu RNAs, and B2 RNAs were retrieved from UCSC Table Browser (https://genome.ucsc.edu/cgi-bin/hgTables); using GENCODE V23, GENCODE V25, and RepeatMasker (as of Jan 2021), with subsequent filtering for specific families of elements. snoRNA annotations were retrieved from Ensembl/ Biomart (as of Feb 2023). The number of lines in the intersected bed files (corresponding to the number of reads which occur within a respective annotation file) is taken as a percentage of the total number of reads present in the pre-intersection bed file, thus giving a proportion of reads which fall into the category represented by each annotation. Transcriptional Start Site (TSS) annotation is based on the Eponine annotation [110]. Models of read distribution around the TSS of various genomic elements were performed using the Babraham NGS analysis suite Seqmonk 1.38.2 (https://www.bioinformatics.babraham.ac.uk/projects/seqmonk/). For rRNA annotation, the Seqmonk default annotation data tracks were used. In brief, we constructed metagene models around a hypothetical set of genomic points, such as the transcription start site and subsequently plotted the distribution of read counts from all contributing elements for each position. Then the numbers of reads at the same strand with the element around each different TSS were calculated and attributed to defined points in the model. Relative density or cumulative distributions at the metagene plots were generated using Seqmonk.

### Sequins analysis

The Sequins RNA Mix B was spiked into the total RNA sample to assess the transcriptome complexity from both the standard and NERD-seq preparations and analyzed using the Anaquin toolkit (3.23.0) as described before [64]. Mapping for the sequin reads was performed using minimap2 under the following parameters: -ax splice -uf –eqx, and a modified mouse genome reference whereby the Ensembl GRCh39 release was concatenated with the RNA sequins mix B pseudochromosomes. RNA read abundances were calculated and compared to known abundances for these RNAs. Sashimi plots were produced using the IGV gene viewer [111].

### RNA modification analysis

The Xpore tool [95] was compiled into a nextflow script and modified to report Student’s *t*-test *p*-values from the Nanopolish output into the final “diffmod” table output. Reads were mapped to the ensembl GRCh38 cDNA transcriptome using recommended parameters. The cerebral cortex human total RNA standard and Nerd-seq datasets were compared to the 7sk IVT dataset.AQ. 

## Supplementary Information

Additional file 1:  Includes supplementary figures Fig S1—Fig S24. A short description of the content of these figures is provided at the first two pages of the file.

Additional file 2:  Includes a supplementary table featuring locations from consensus sequence of sequence present in L1Md_t through BLAT.

Additional file 3:  Peer review history.

## Data Availability

NERD-seq data have been deposited to SRA with access number PRJNA1145394 [112] for human data and PRJNA1145116 for mouse data [113]. The external dataset from Sessegolo et al. [61] was accessed from the European Nucleotide Archive, accession: PRJEB27590 [114] (https://www.genoscope.cns.fr/ont_mouse_rna/datasets_RNA_LR.html). Samples Brain C1 and C2 were used for comparison. The external dataset from Cheng et al. [115] can be accessed from GEO accession: GSE149243 [115].
