# Author Correction: NERD-seq: a novel approach of Nanopore direct RNA sequencing that expands representation of non-coding RNAs

**DOI:** 10.1186/s13059-024-03395-4

**Published:** 2024-09-26

**Authors:** Luke Saville, Li Wu, Jemaneh Habtewold, Yubo Cheng, Babita Gollen, Liam Mitchell, Matthew Stuart-Edwards, Travis Haight, Majid Mohajerani, Athanasios Zovoilis

**Affiliations:** 1 Department of Biochemistry and Medical Genetics, University of Manitoba, Winnipeg, MB R3E3N4 Canada; 2 Paul Albrechtsen Research Institute, CCMB, Winnipeg, MB R3E3N4 Canada; 3 Southern Alberta Genome Sciences Centre, University of Lethbridge, Lethbridge, AB T1K3M4 Canada; 4 Canadian Centre for Behavioral Neuroscience, University of Lethbridge, Lethbridge, AB T1K3M4 Canada

**Correction:** **Genome Biol 25, 233 (2024)**

**https://doi.org/10.1186/s13059-024-03375-8**

Following publication of the original article [1], the authors identified two typos in affiliation 3 and 4. The incorrect and correct affiliations are given below.

Incorrect: ^3^ Southern Alberta Genome Sciences Centre, Lethbridge, AB, T1K3M4, Canada

Correct: ^3^ Southern Alberta Genome Sciences Centre, University of Lethbridge, Lethbridge, AB, T1K3M4, Canada

Incorrect: ^4^ Canadian Centre for Behavioral Neuroscience, Lethbridge, AB, T1K3M4, Canada

Correct: ^4^ Canadian Centre for Behavioral Neuroscience, University of Lethbridge, Lethbridge, AB, T1K3M4, Canada

The original article [1] is corrected.
