# Supplementary material for: NERD-seq: a novel approach of Nanopore direct RNA sequencing that expands representation of non-coding RNAs
Source: Genome Biol. 2024 Aug 28;25:233. doi: 10.1186/s13059-024-03375-8 (PMC11351768; doi:10.1186/s13059-024-03375-8)
Supplement: Supplementary file 1 — Additional file 1: Includes supplementary figures Fig S1—Fig S24. A short description of the content of these figures is provided at the first two pages of the file. [file 13059_2024_3375_MOESM1_ESM.pdf]

NERD-seq: A novel approach of Nanopore direct RNA sequencing that expands representation of non-coding RNAs

Luke Saville, Li Wu, Jemaneh Habtewold, Babita Gollen, Liam Mitchell, Matthew Stuart-Edwards, Travis Haight, Majid Mohajerani, Athanasios Zovoilis

Additional file 1

(Supplementary Figures)

CONTENTS

Fig S1. Use of Omniamp polymerase in the NERD-seq protocol produces similar distributions for polyA RNAs with those using GspSSD2.0.

Fig S2. Omniamp polymerase produces similar distributions of gene families: snoRNAs, snRNAs, scRNAs and srpRNAs with NERD-seq protocol.

Fig S3. Use of Omniamp polymerase in the NERD-seq protocol produces similar distributions with those using GspSSD2.0 for the following RNA families: tRNAs and rRNAs/rRFs.

Fig S4. Use of Omniamp polymerase in the NERD-seq protocol produces similar distributions with those using GspSSD2.0 for LINE1 derived RNAs.

Fig S5. Optimizing NERD-seq library conditions.

Fig S6. Validation of results on snoRNAs, snRNAs, scRNAs and srpRNAs comparing NERD-seq data with data from an external dataset that has been generated by the standard direct RNA-seq approach.

Fig S7. Validation of results on tRNAs and rRFs comparing NERD-seq data with data from the external dataset that has been generated by the standard direct RNA-seq approach.

Fig S8. NERD-seq can detect poly(A) ncRNAs.

Fig S9. NERD-seq can detect mRNAs and poly(A) ncRNAs in human cerebral cortex.

Fig S10. RNA sequins mix B analysis in NERD-seq vs Standard direct RNA-seq approach.

Fig S11. Different mapping presets produce differing profiles in mRNAs from NERD-seq.

Fig S12. NERD-seq reliably enriches snoRNAs, similarly to Illumina short RNA libraries.

Fig S13. NERD-seq reliably enriches snRNAs, similarly to Illumina short RNA libraries.

Fig S14. NERD-seq reliably enriches scRNAs, similarly to Illumina short RNA libraries.

Fig S15. NERD-seq reliably enriches srpRNAs, similarly to Illumina short RNA libraries.

Fig S16. NERD-seq enriches ncRNAs in human cerebral cortex.

Fig S17. NERD-seq reliably enriches tRNAs, similarly to Illumina short RNA libraries.

Fig S18. NERD-seq can detect tRNAs and rRNAs in human cerebral cortex.

Fig S19. NERD-seq reliably enriches rRNAs, similarly to Illumina short RNA libraries.

Fig S20. Small RNAQC plot shows NERD-seq is more replicable than the standard approach in the 25-50bp range.

Fig S21. LSU-rRNA_HSA reads are enriched in the mouse hippocampus samples.

Fig S22. L1Md_T mapped reads (mm10 – chr13: 9,832,020-9,838,665) in NERD-seq samples aligns with high similarity to the rDNA repeating gene (Genbank: BK000964.3).

Fig S23. Known pseudouridine sites in ncRNAs are detectable using NERD-seq.

Fig S24. NERD-seq replicates patterns of enrichment with SQK_RNA004 Nanopore chemistry.

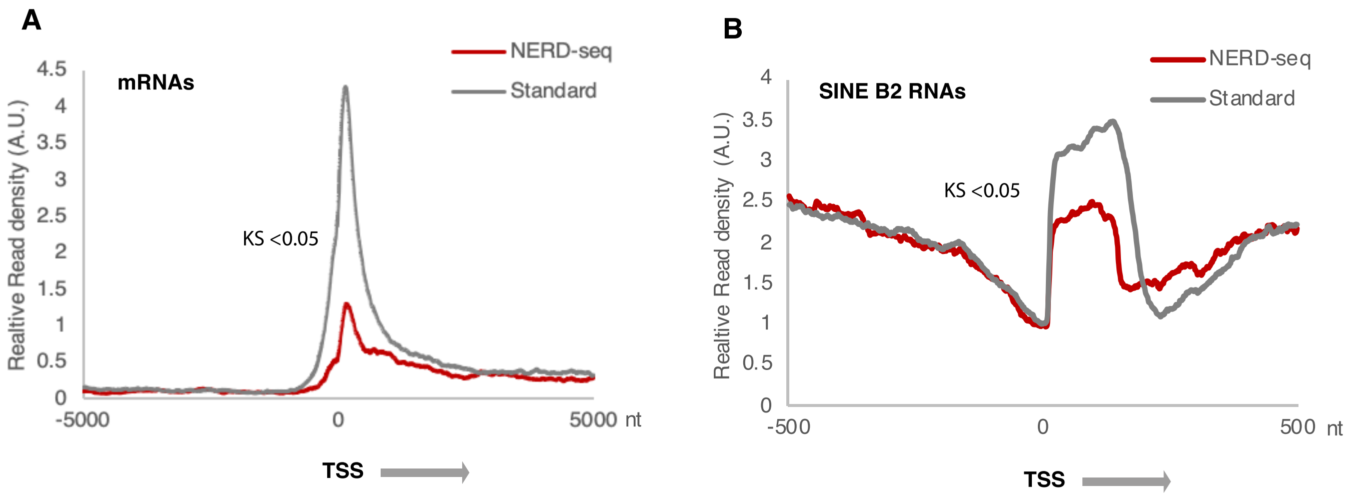

A
mRNAs
NERD-seq
Standard
Realtive Read density (A.U.)
4.5
4
3.5
3
2.5
2
1.5
1
0.5
0
KS <0.05
-5000
0
5000 nt
TSS
B
SINE B2 RNAs
NERD-seq
Standard
Realtive Read density (A.U.)
4
3.5
3
2.5
2
1.5
1
0.5
0
KS <0.05
-500
0
500 nt
TSS

Fig S1

Fig S1. Use of Omniamp polymerase in the NERD-seq protocol produces similar distributions for polyA RNAs with those using GspSSD2.0.

Metagene plots produced on data from RNA sequencing using the standard direct RNA-seq and a NERD-seq approach with a different LAMP enzyme than GspSSD2.0, called Omniamp polymerase.

(A) Relative read density distribution around the Transcription Start Site (TSS) of known genes for standard and NERD-seq. A metagene model has been constructed by aligning all known genes at their Transcription Start Site (as estimated by the use of Eponine at mm10 (53)) on the same strand. Distances at X axis correspond to absolute distance (in nucleotides; nt) 500nt upstream (left) and downstream (right) from TSS. The arrow next to TSS depicts the direction of transcription. Read density is calculated by dividing the number of reads aligning to each position, divided to the total number of reads and elements (genes) that construct the metagene. KS: Kolmogorov–Smirnov test (KS) < 0.05 for the comparison between the two distributions. Both sequencing types were aligned using minimap2 with splice aware mapping enabled.

(B) Relative read density distribution around the Transcription Start Site (TSS) of SINE B2 RNAs (repeat masker mm10) for standard and NERD-seq. KS: Kolmogorov–Smirnov test (KS) < 0.05 for the comparison between the two distributions. Both sequencing types were aligned using minimap2 with the sr preset enabled. Line plot depicts 500nt upstream (-500) and downstream (500) of TSS (0nt).

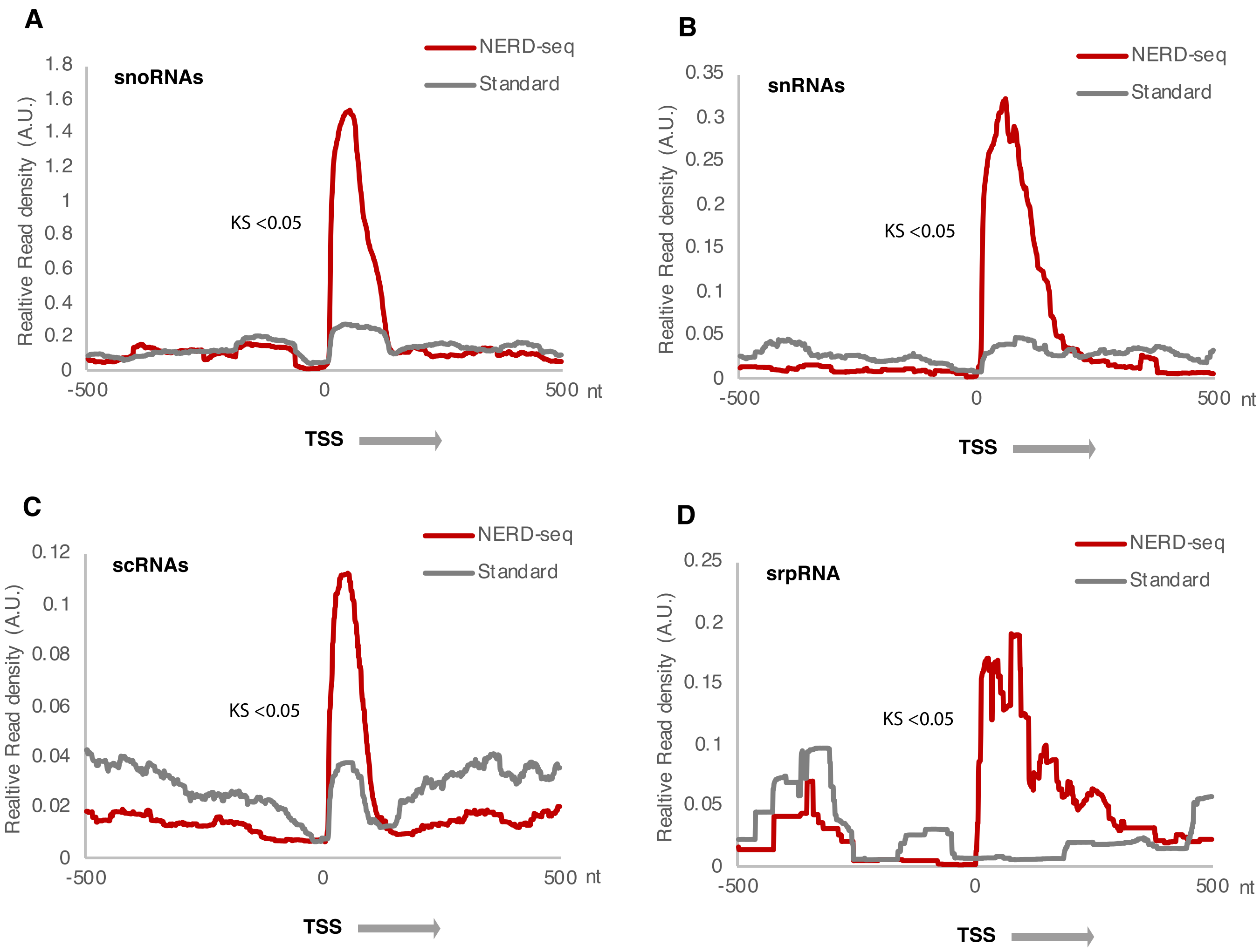
A
snoRNAs
NERD-seq
Standard
Realtive Read density (A.U.)
1.8
1.6
1.4
1.2
1
0.8
0.6
0.4
0.2
0
KS <0.05
-500
0
500 nt
TSS
B
snRNAs
NERD-seq
Standard
Realtive Read density (A.U.)
0.35
0.3
0.25
0.2
0.15
0.1
0.05
0
KS <0.05
-500
0
500 nt
TSS
C
scRNAs
NERD-seq
Standard
Realtive Read density (A.U.)
0.12
0.1
0.08
0.06
0.04
0.02
0
KS <0.05
-500
0
500 nt
TSS
D
srpRNA
NERD-seq
Standard
Realtive Read density (A.U.)
0.25
0.2
0.15
0.1
0.05
0
KS <0.05
-500
0
500 nt
TSS

Fig S2

Fig S2. Omniamp polymerase produces similar distributions of gene families: snoRNAs, snRNAs, scRNAs and srpRNAs with NERD-seq protocol.

Metagene plots produced on data from RNA sequencing using the standard and a modified NERD-seq approach with a different LAMP enzyme, called Omniamp polymerase.

(A) Relative read density distribution around the Transcription Start Site (TSS) of snoRNAs for external standard and NERD-seq. X, Y axis and KS-test as in Figure 3.

(B) Relative read density distribution around the Transcription Start Site (TSS) of snRNAs.

(C) Relative read density distribution around the Transcription Start Site (TSS) of scRNAs.

(D) Relative read density distribution around the Transcription Start Site (TSS) of srpRNA.

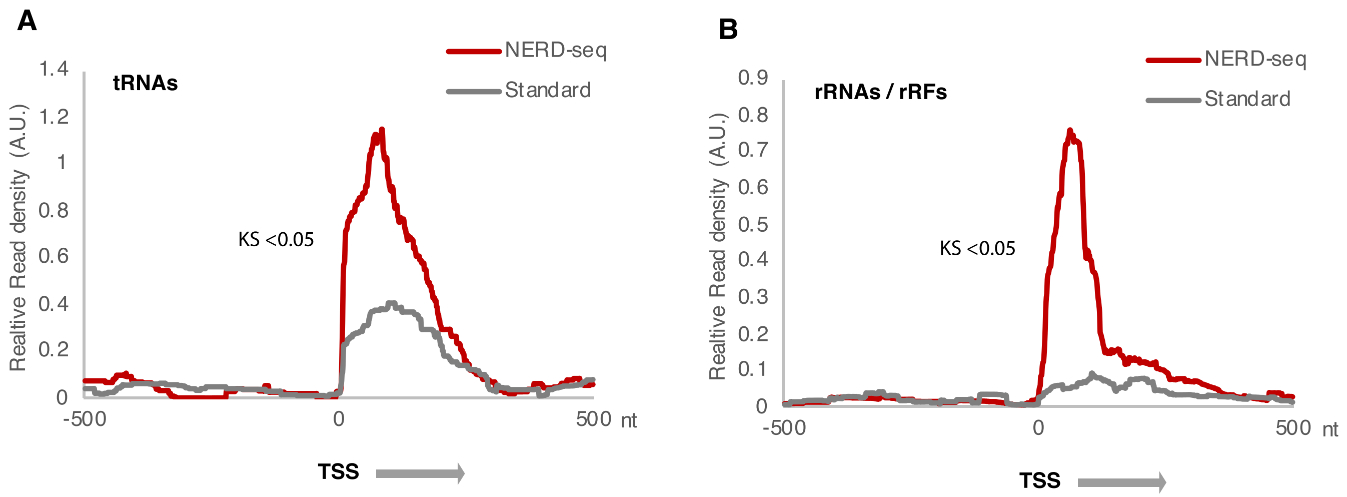
A
tRNAs
NERD-seq
Standard
Realtive Read density (A.U.)
1.4
1.2
1
0.8
0.6
0.4
0.2
0
KS <0.05
-500
0
500 nt
TSS
B
rRNAs / rRFs
NERD-seq
Standard
Realtive Read density (A.U.)
0.9
0.8
0.7
0.6
0.5
0.4
0.3
0.2
0.1
0
KS <0.05
-500
0
500 nt
TSS

Fig S3

Fig S3. Use of Omniamp polymerase in the NERD-seq protocol produces similar distributions with those using GspSSD2.0 for the following RNA families: tRNAs and rRNAs/rRFs.

Metagene plots produced on data using the standard direct RNA-seq and a NERD-seq approach with the Omniamp polymerase.

(A) Relative read density distribution around the Transcription Start Site (TSS) of known tRNAs (mm10) for external standard and NERD-seq. X, Y axis and KS-test as in Figure 3.

(B) Relative read density distribution around the Transcription Start Site (TSS) of rRNAs (mm10).

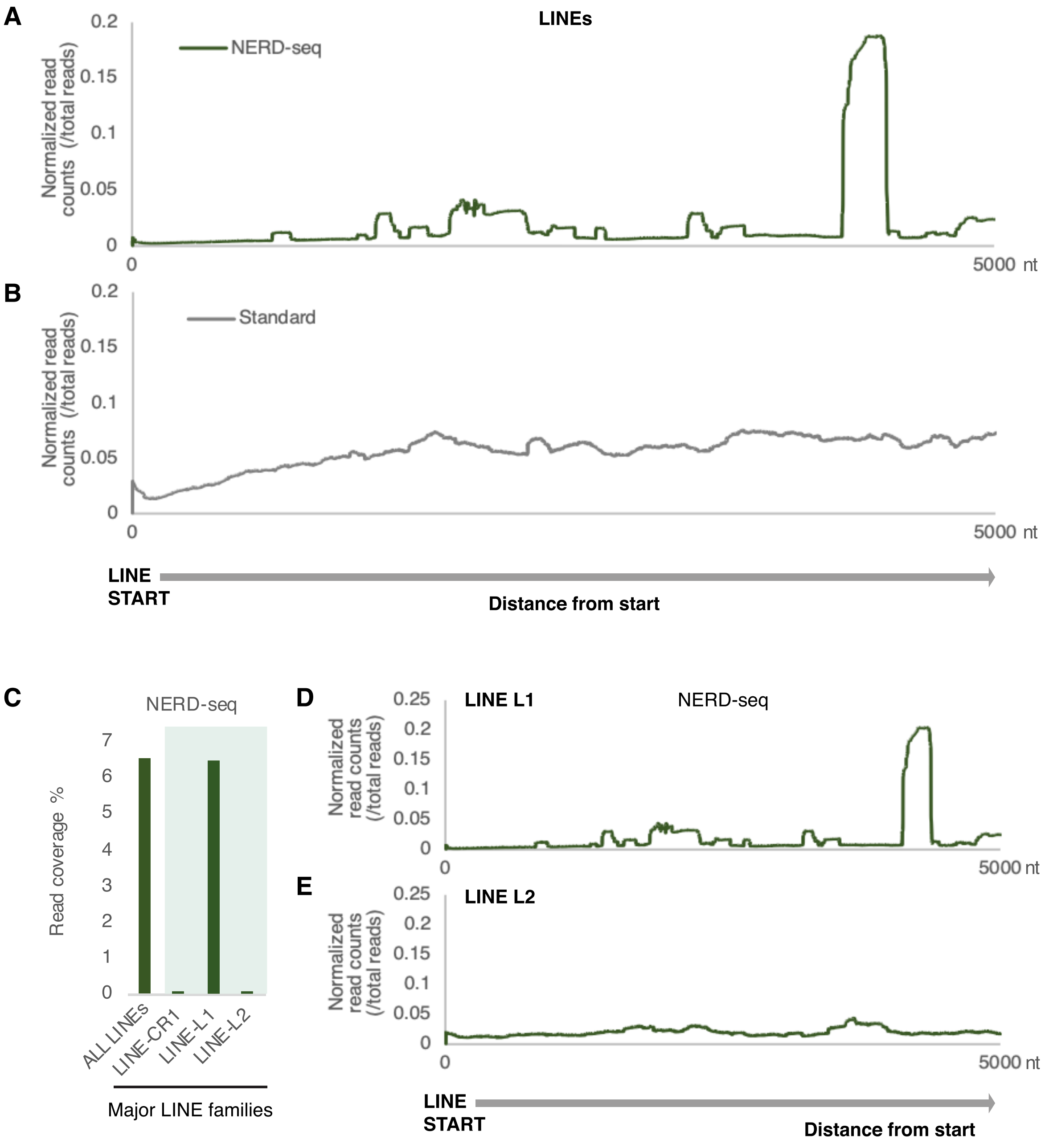
A
LINEs
NERD-seq
Normalized read counts (/total reads)
0.2
0.15
0.1
0.05
0
0
5000 nt
B
Standard
Normalized read counts (/total reads)
0.2
0.15
0.1
0.05
0
0
5000 nt
LINE START
Distance from start
C
NERD-seq
Read coverage %
7
6
5
4
3
2
1
0
ALL LINEs
LINE-CR1
LINE-L1
LINE-L2
Major LINE families
D
LINE L1
NERD-seq
Normalized read counts (/total reads)
0.25
0.2
0.15
0.1
0.05
0
0
5000 nt
E
LINE L2
Normalized read counts (/total reads)
0.25
0.2
0.15
0.1
0.05
0
0
5000 nt
LINE START
Distance from start

Fig S4

Fig S4. Use of Omniamp polymerase in the NERD-seq protocol produces similar distributions with those using GspSSD2.0 for LINE1 derived RNAs. Metagene plots produced on data using the standard direct RNA-seq and a NERD-seq approach with the Omniamp polymerase.

(A) Normalized counts of reads from NERD-seq mapped across the first 5000nt of a metagene constructed by all known LINE elements (repeat masker UCSC mm10). The metagene model has been constructed by aligning all known LINEs at their start site (base #1 in each element's DNA sequence). Distances at X axis correspond to absolute distance (in nucleotides; nt) 5000nt downstream from the start. The arrow below the graph corresponds to the sense direction of the elements. Normalized read counts per position are calculated by dividing the number of reads aligning to each position to the total number of reads.

(B) Same as in (A) but for standard direct RNA-seq reads.

(C) Coverage (percentage) across all LINEs and across the three LINE subfamilies for NERD-seq reads. The three LINE subfamilies with most aligned reads have been selected and are depicted here.

(D) Per million reads normalized counts of reads from NERD-seq mapped across the first 5000nt of a metagene constructed as in (A) but by all known LINE L1 family elements.

(E) Per million reads normalized counts of reads from NERD-seq mapped across the first 5000nt of a metagene constructed as in (A) but by all known LINE L2 family elements.

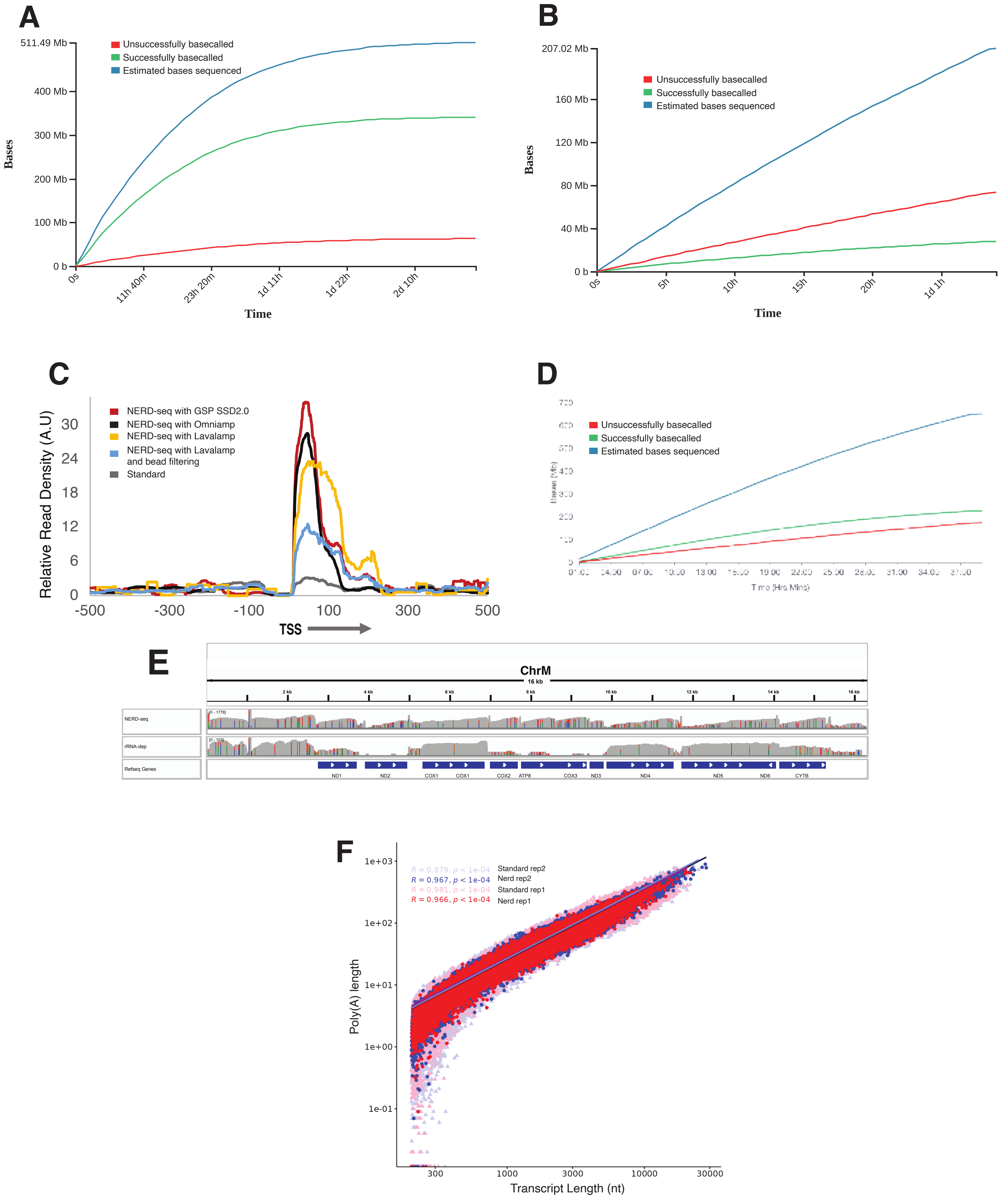
A
511.49 Mb
400 Mb
300 Mb
200 Mb
100 Mb
0 b
Bases
Unsuccessfully basecalled
Successfully basecalled
Estimated bases sequenced
0s
11h 40m
23h 20m
1d 11h
1d 22h
2d 10h
Time
B
207.02 Mb
160 Mb
120 Mb
80 Mb
40 Mb
0 b
Bases
Unsuccessfully basecalled
Successfully basecalled
Estimated bases sequenced
0s
5h
10h
15h
20h
1d 1h
Time
C
Relative Read Density (A.U)
30
24
18
12
6
0
NERD-seq with GSP SSD2.0
NERD-seq with Omniamp
NERD-seq with Lavalamp
NERD-seq with Lavalamp and bead filtering
Standard
-500
-300
-100
100
300
500
TSS
D
Unsuccessfully basecalled
Successfully basecalled
Estimated bases sequenced
E
ChrM
16 kb
2 kb
4 kb
6 kb
8 kb
10 kb
12 kb
14 kb
16 kb
NERD-seq
rRNA-dep
Refseq Genes
ND1
ND2
COX1
COX1
COX2
ATP8
COX3
ND3
ND4
ND5
ND6
CYTB
F
R = 0.979, p < 1e-04 Standard rep2
R = 0.967, p < 1e-04 Nerd rep2
R = 0.981, p < 1e-04 Standard rep1
R = 0.966, p < 1e-04 Nerd rep1
1e+03
1e+02
1e+01
1e+00
1e-01
Poly(A) length
300
1000
3000
10000
30000
Transcript Length (nt)

Fig S5

Fig S5. Optimizing NERD-seq library conditions.

In an effort to find the enzyme and conditions most suitable for enriching short ncRNAs in our library, we screened multiple enzymes and protocol parameters we felt matched the criteria for resolving highly structured RNAs so they could be linearized during the sequencing process.

A) Minknow output of Bases produced over the library sequencing process of a NERD-seq library.

B) Minknow output of Bases produced over the library sequencing process from of a library replicating the NERD-seq protocol conditions using the Lavalamp enzyme.

C) Metagene plot, depicting relative read density distribution around the Transcription Start Site (TSS) of snoRNAs for varying enzymatic conditions, including the use of GSP SSD2.0 (the one used in NERD-seq), Omniamp which also enriched snoRNAs but became commercially unavailable and Lavalamp which seems to optimize for shorter reads. As well, we attempted to bead clean (2.2X) the Lavalamp treated samples to reduce the concentration of very small RNAs to attempt to improve the pool of first strand synthesized RNAs of longer lengths, but this failed to improve enrichment of RNAs like those belonging to the snoRNA family.

D) Minknow output of Bases produced over the library sequencing process of a modified NERD-seq library using rRNA depletion and polyadenylation capture to attempt to capture all reads.

E) IGV viewer image of the mouse mitochondrial chromosome where bars exhibit read coverage over the annotation. The coverage view thresholds remained default (range depicted in top left of coverage window). NERD-seq library (top coverage window) has a threshold between 0-1,778 and the rRNA depleted and subsequently polyadenylated library (as in Figure S5-D) has a threshold between 0-103.

F) Poly(A) tail lengths are correlated to transcript length. Scatterplot depicting the transcript lengthof reads against their poly(A) tail length, calculated using Nanopolish as suggested here: https://nanopolish.readthedocs.io/en/latest/quickstart_polya.html. Pearson coefficient and Pvalue produced using the stat_cor function.

From the data presented in Fig S5, we demonstrated that the GSP SSD2.0 enzyme and the library parameters used in the NERD-seq protocol are the optimal conditions needed to enrich short ncRNAs at the same time as sequencing mRNAs.

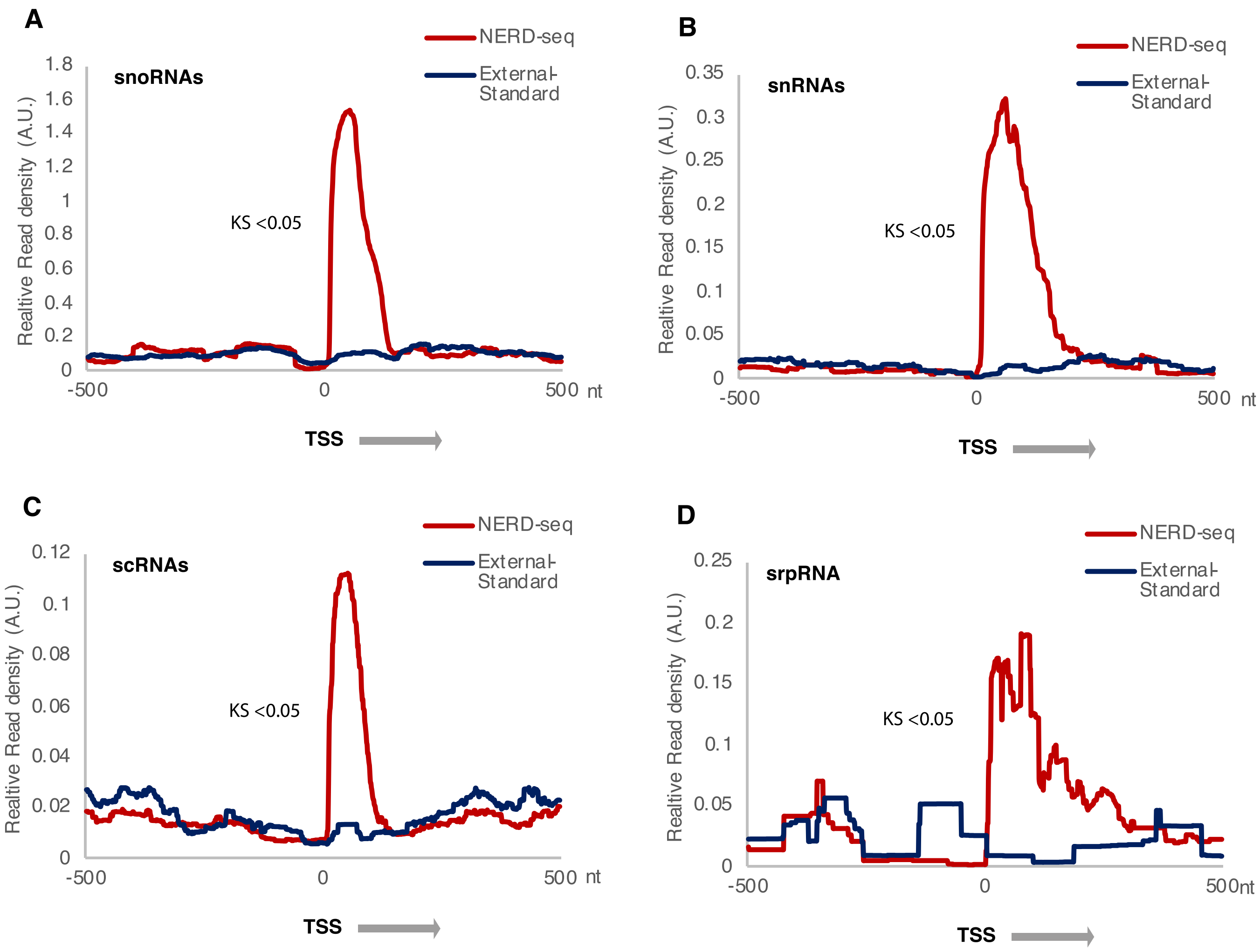

A
snoRNAs
NERD-seq
External Standard
KS <0.05
Realtive Read density (A.U.)
1.8
1.6
1.4
1.2
1
0.8
0.6
0.4
0.2
0
-500
0
500 nt
TSS
B
snRNAs
NERD-seq
External Standard
KS <0.05
Realtive Read density (A.U.)
0.35
0.3
0.25
0.2
0.15
0.1
0.05
0
-500
0
500 nt
TSS
C
scRNAs
NERD-seq
External Standard
KS <0.05
Realtive Read density (A.U.)
0.12
0.1
0.08
0.06
0.04
0.02
0
-500
0
500 nt
TSS
D
srpRNA
NERD-seq
External Standard
KS <0.05
Realtive Read density (A.U.)
0.25
0.2
0.15
0.1
0.05
0
-500
0
500nt
TSS

Fig S6

Fig S6. Validation of results on snoRNAs, snRNAs, scRNAs and srpRNAs comparing NERD-seq data with data from an external dataset that has been generated by the standard direct RNA-seq approach.

(A) Relative read density distribution around the Transcription Start Site (TSS) of snoRNAs for external standard and NERD-seq. X, Y axis and KS-test as in Figure 3.

(B) Relative read density distribution around the Transcription Start Site (TSS) of snRNAs.

(C) Relative read density distribution around the Transcription Start Site (TSS) of scRNAs.

(D) Relative read density distribution around the Transcription Start Site (TSS) of srpRNA.

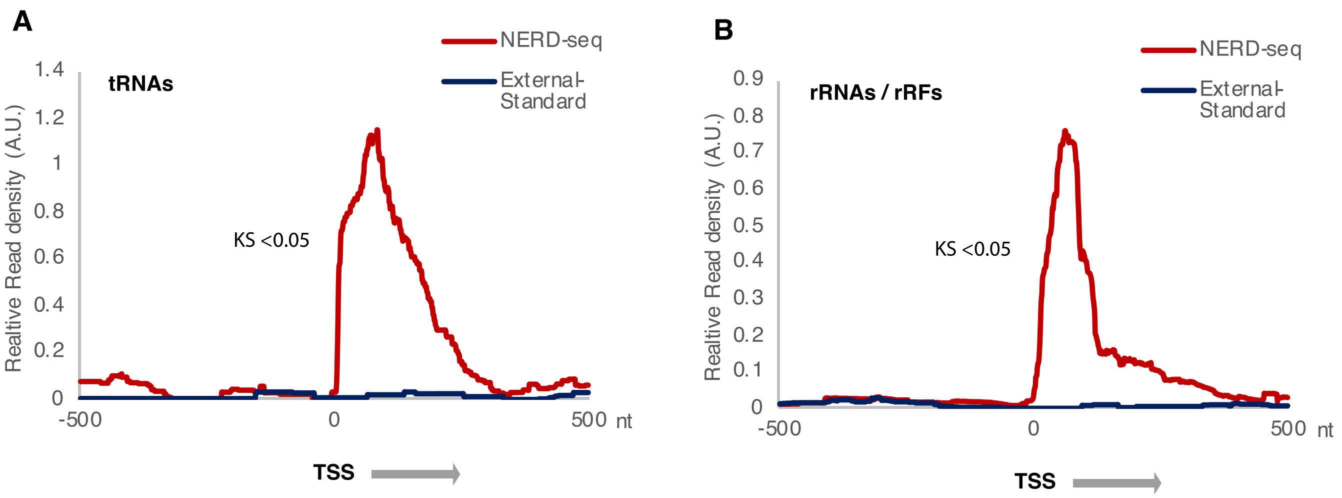
A
NERD-seq
External-Standard
tRNAs
Realtive Read density (A.U.)
1.4
1.2
1
0.8
0.6
0.4
0.2
0
KS <0.05
-500
0
500 nt
TSS
B
NERD-seq
External-Standard
rRNAs / rRFs
Realtive Read density (A.U.)
0.9
0.8
0.7
0.6
0.5
0.4
0.3
0.2
0.1
0
KS <0.05
-500
0
500 nt
TSS

Fig S7

Fig S7. Validation of results on tRNAs and rRFs comparing NERD-seq data with data from the external dataset that has been generated by the standard direct RNA-seq approach.

(A) Relative read density distribution around the Transcription Start Site (TSS) of known tRNAs (mm10) for external standard and NERD-seq. X, Y axis and KS-test as in Figure 3.

(B) Relative read density distribution around the Transcription Start Site (TSS) of rRNAs (mm10).

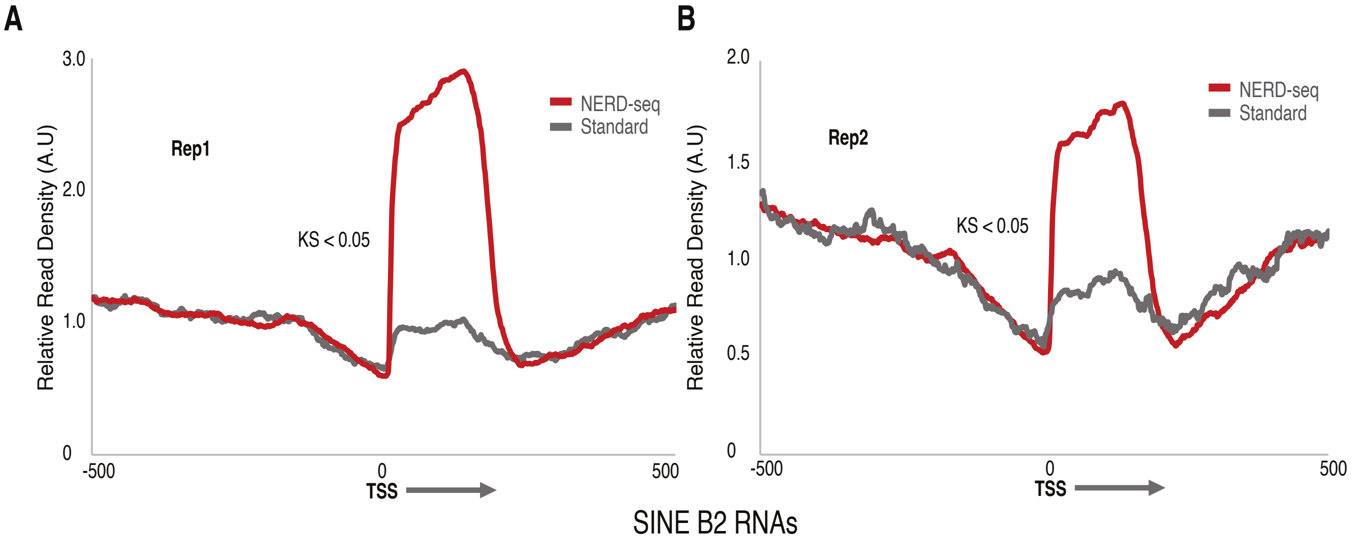
A
B
Relative Read Density (A.U)
3.0
2.0
1.0
0
Rep1
KS < 0.05
NERD-seq
Standard
-500
0
500
TSS
Relative Read Density (A.U)
2.0
1.5
1.0
0.5
0
Rep2
KS < 0.05
NERD-seq
Standard
-500
0
500
TSS
SINE B2 RNAs

Fig S8

Fig S8. NERD-seq can detect poly(A) ncRNAs.

Relative read density distribution around the Transcription Start Site (TSS) of SINE B2 RNAs (repeat masker mm10) for standard and NERD-seq for two biological replicates (Rep1 in (A) and Rep2 in (B)). KS: Kolmogorov–Smirnov test (KS) < 0.05 for the comparison between the two distributions. Both sequencing types were aligned using minimap2 with the sr preset enabled. Line plot depicts 500nt upstream (-500) and downstream (500) of TSS (0nt).

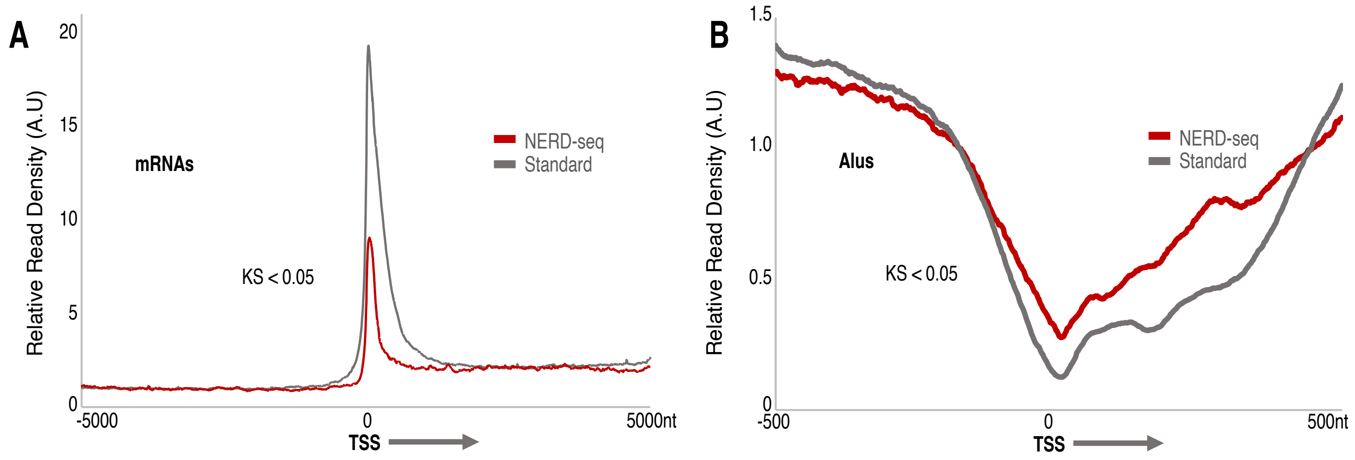

A
B
mRNAs
Alus
KS < 0.05
KS < 0.05
NERD-seq
Standard
Relative Read Density (A.U)
20
15
10
5
0
1.5
1.0
0.5
-5000
0
5000nt
-500
500nt
TSS

Fig S9

Fig S9. NERD-seq can detect mRNAs and poly(A) ncRNAs in human cerebral cortex.

(A) Relative read density distribution around the Transcription Start Site (TSS) of known genes for standard and NERD-seq. A metagene model has been constructed by aligning all known genes at their Transcription Start Site (as estimated by the use of Eponine at mm10 (53)) on the same strand. Both sequencing types were aligned using minimap2 with splice aware mapping enabled.

(B) Relative read density distribution around the Transcription Start Site (TSS) of SINE Alu RNAs (repeat masker mm10) for standard and NERD-seq. KS: Kolmogorov–Smirnov test (KS) < 0.05 for the comparison between the two distributions. Both sequencing types were aligned using minimap2 with the sr preset enabled. Line plot depicts 500nt upstream (-500) and downstream (500) of TSS (0nt).

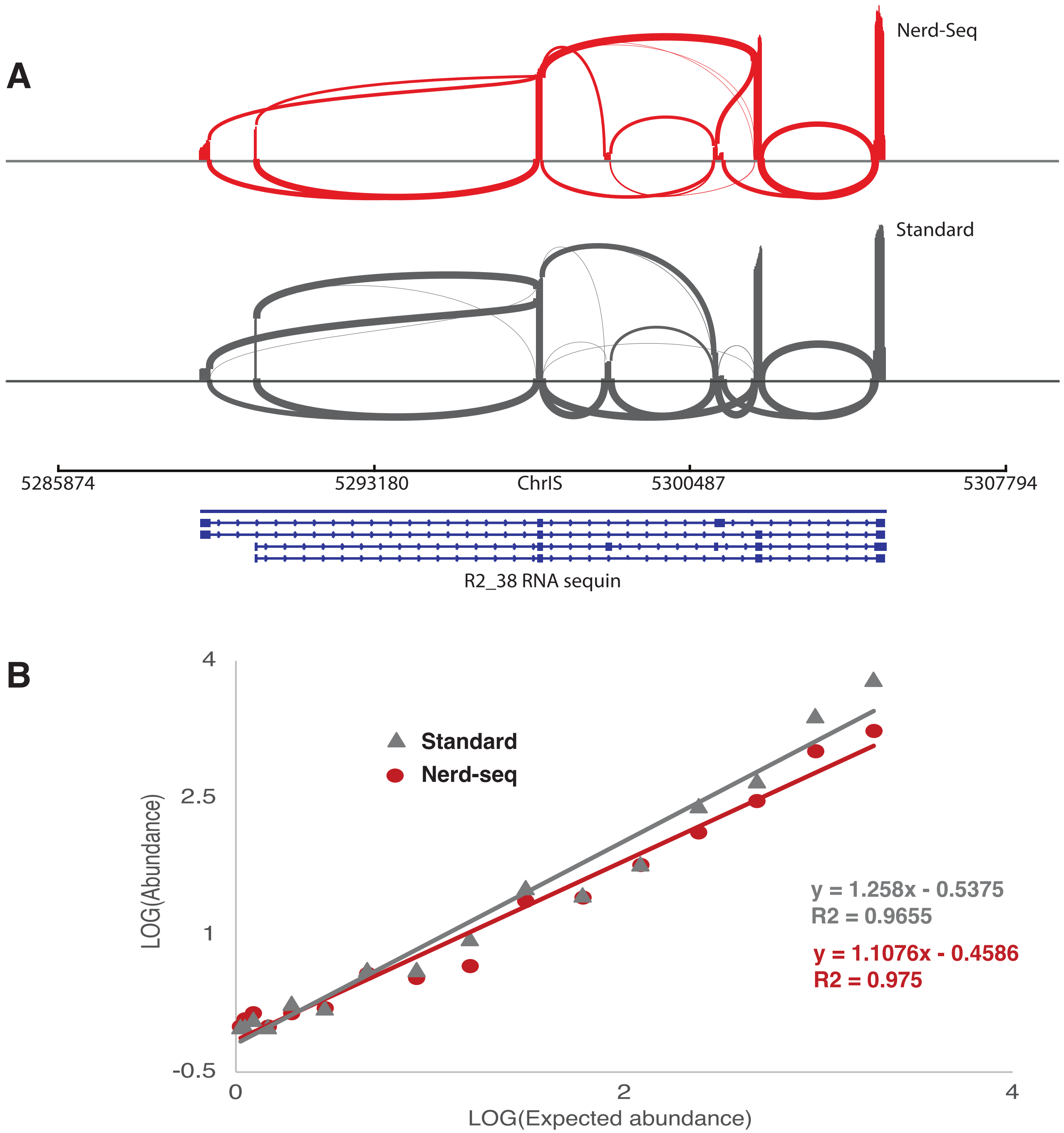
A
Nerd-Seq
Standard
5285874
5293180
ChrIS
5300487
5307794
R2_38 RNA sequin
B
4
2.5
1
-0.5
LOG(Abundance)
Standard
Nerd-seq
y = 1.258x - 0.5375
R2 = 0.9655
y = 1.1076x - 0.4586
R2 = 0.975
0
2
4
LOG(Expected abundance)

Fig S10

Fig S10. RNA sequins mix B analysis in NERD-seq vs Standard direct RNA-seq approach.
(A) Sashimi plot of R2_38 synthetic gene spiked in before library preparation. Splicing patterns are well reproduced between NERD-seq (top) and Standard (bottom) despite lowered coverage. IGV depiction of the R2_38 RNA sequin gene, including expected introns and exons is depicted in blue. Region depicted is ChrIS: 5285874-5307794 as an assembly concatenated with the mm10 assembly.
(B) Anaquin software pipeline output of gene quantification by expected abundance (X) vs measured abundance (Y). Regression equations and Rsquared values are depicted in the lower right.

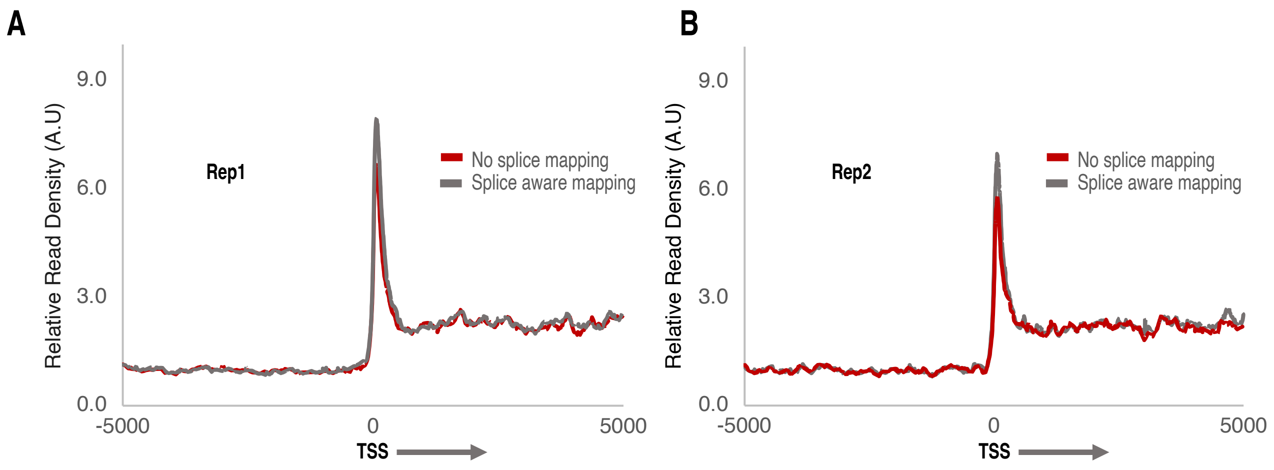
A
Rep1
No splice mapping
Splice aware mapping
Relative Read Density (A.U)
9.0
6.0
3.0
0.0
-5000
0
5000
TSS
B
Rep2
No splice mapping
Splice aware mapping
Relative Read Density (A.U)
9.0
6.0
3.0
0.0
-5000
0
5000
TSS

Fig S11

Fig S11. Different mapping presets produce differing profiles in mRNAs from NERD-seq

Relative read density distribution around the Transcription Start Site (TSS) of known genes for NERD-seq. A metagene model has been constructed by aligning all known genes at their Transcription Start Site (as estimated by the use of Eponine at mm10(53)) on the same strand. Distances at X axis correspond to absolute distance (in nucleotides; nt) 5000nt upstream (left) and downstream (right) from TSS. The arrow next to TSS depicts the direction of transcription. Read density is calculated by dividing the number of reads aligning to each position, divided to the total number of reads and elements (genes) that construct the metagene. Plots juxtapose the minimap2 “splice” preset against the “sr” preset in A-B for biological replicates 1 (A) and 2 (B).

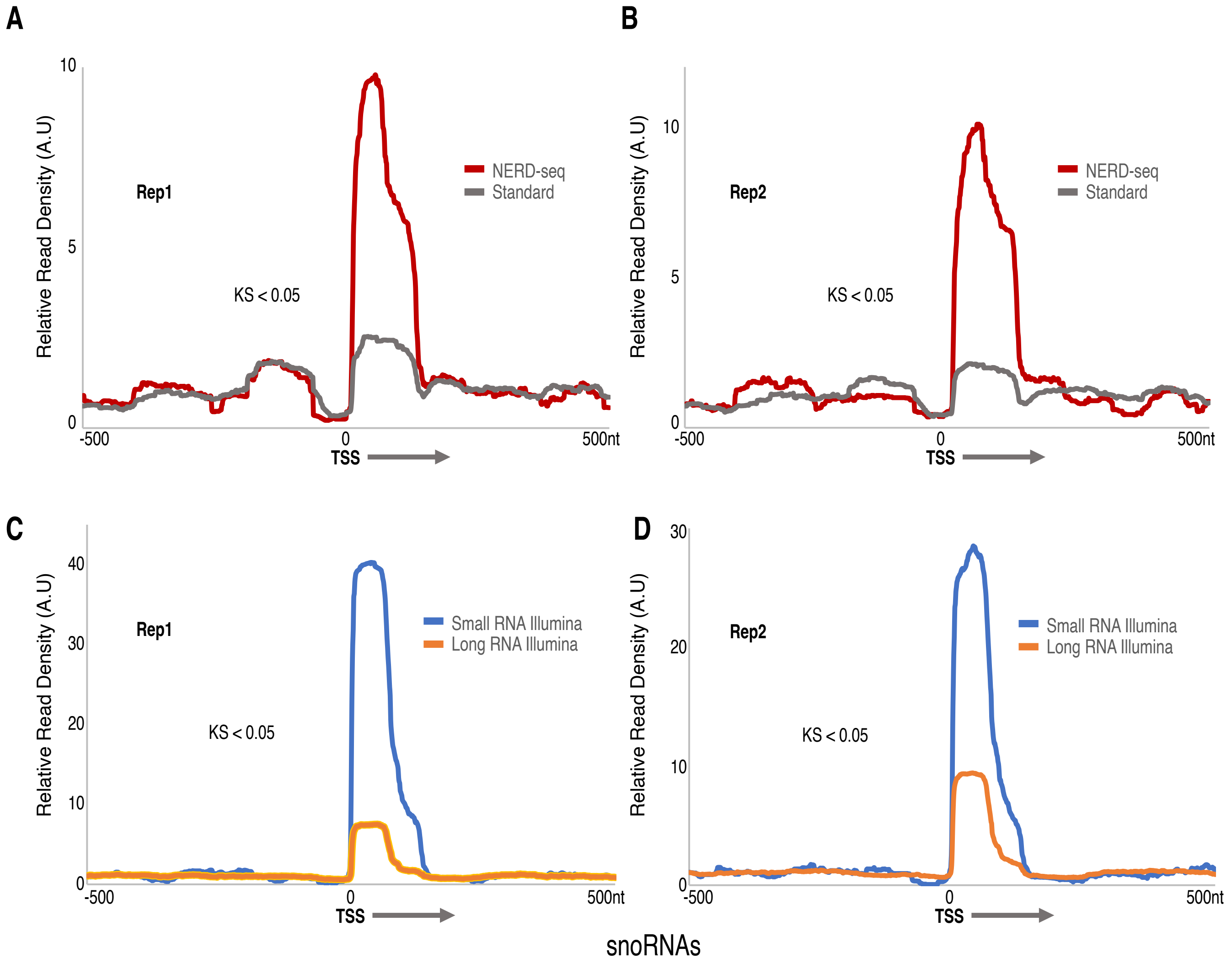
A
B
C
D
Relative Read Density (A.U)
Rep1
Rep2
KS < 0.05
NERD-seq
Standard
Small RNA Illumina
Long RNA Illumina
-500
0
500nt
TSS
snoRNAs

Fig S12

Fig S12. NERD-seq reliably enriches snoRNAs, similarly to Illumina short RNA libraries. Metagene plots depicting relative read density around the transcription start site (TSS) for gene family**:** snoRNA. NERD-seq and standard libraries are compared in A-B with biological replicate 1 (A) and replicate 2 (B). X, Y axis and KS-test as in Figure 3. Similarly, Illumina long and short RNA libraries are compared for relative snoRNA expression levels in C-D with the same biological replicates 1 (C) and 2 (D). X, Y axis and KS-test as in Figure 3.

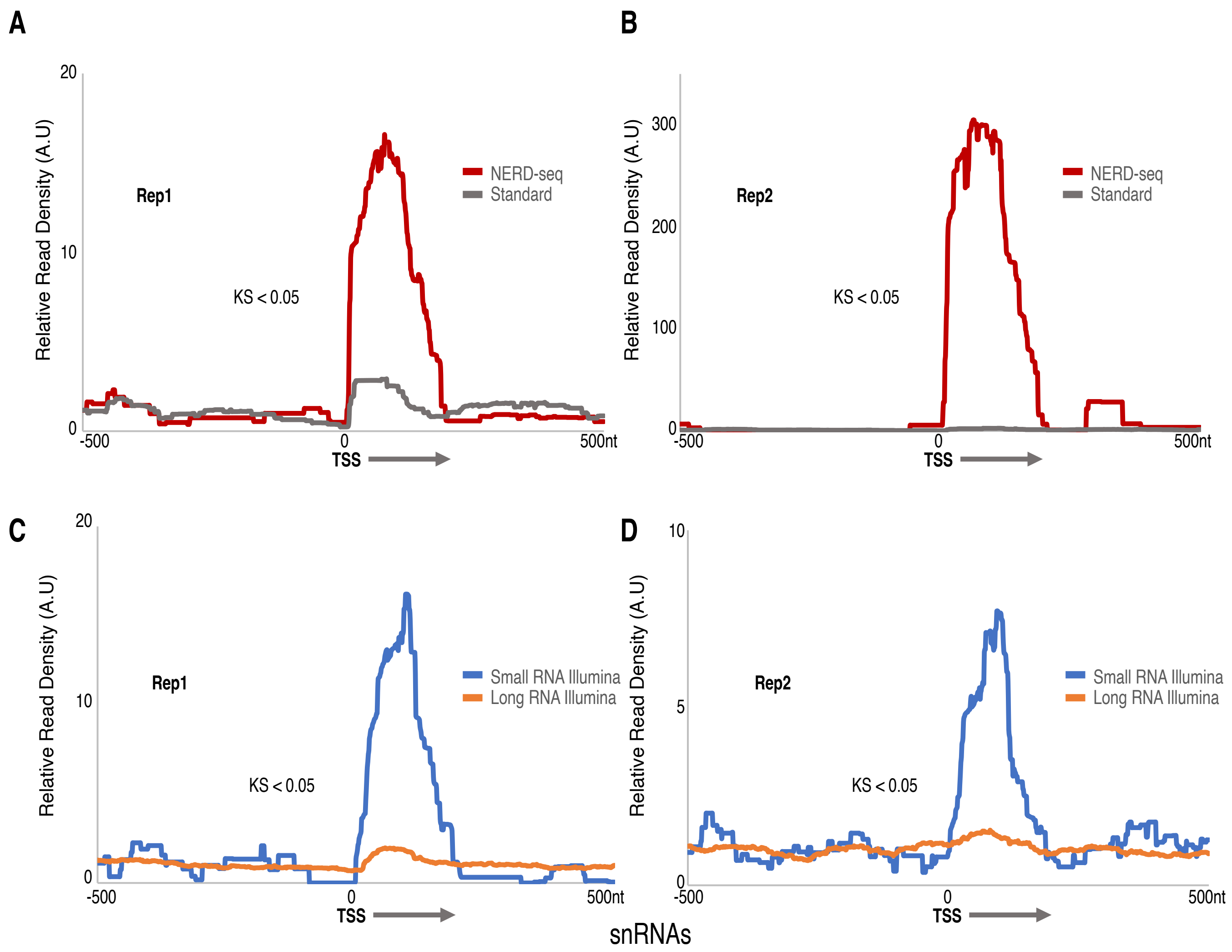

A
Rep1
KS < 0.05
NERD-seq
Standard
Relative Read Density (A.U)
B
Rep2
C
Small RNA Illumina
Long RNA Illumina
D
TSS
-500
0
500nt
snRNAs

Fig S13

Fig S13. NERD-seq reliably enriches snRNAs, similarly to Illumina short RNA libraries. Metagene plots depicting relative read density around the transcription start site (TSS) for gene family: snRNA. NERD-seq and standard libraries are compared in A-B with biological replicate 1 (A) and replicate 2 (B). X, Y axis and KS-test as in Figure 3. Similarly, Illumina long and short RNA libraries are compared for relative snRNA expression levels in C-D with the same biological replicates 1 (C) and 2 (D). X, Y axis and KS-test as in Figure 3.

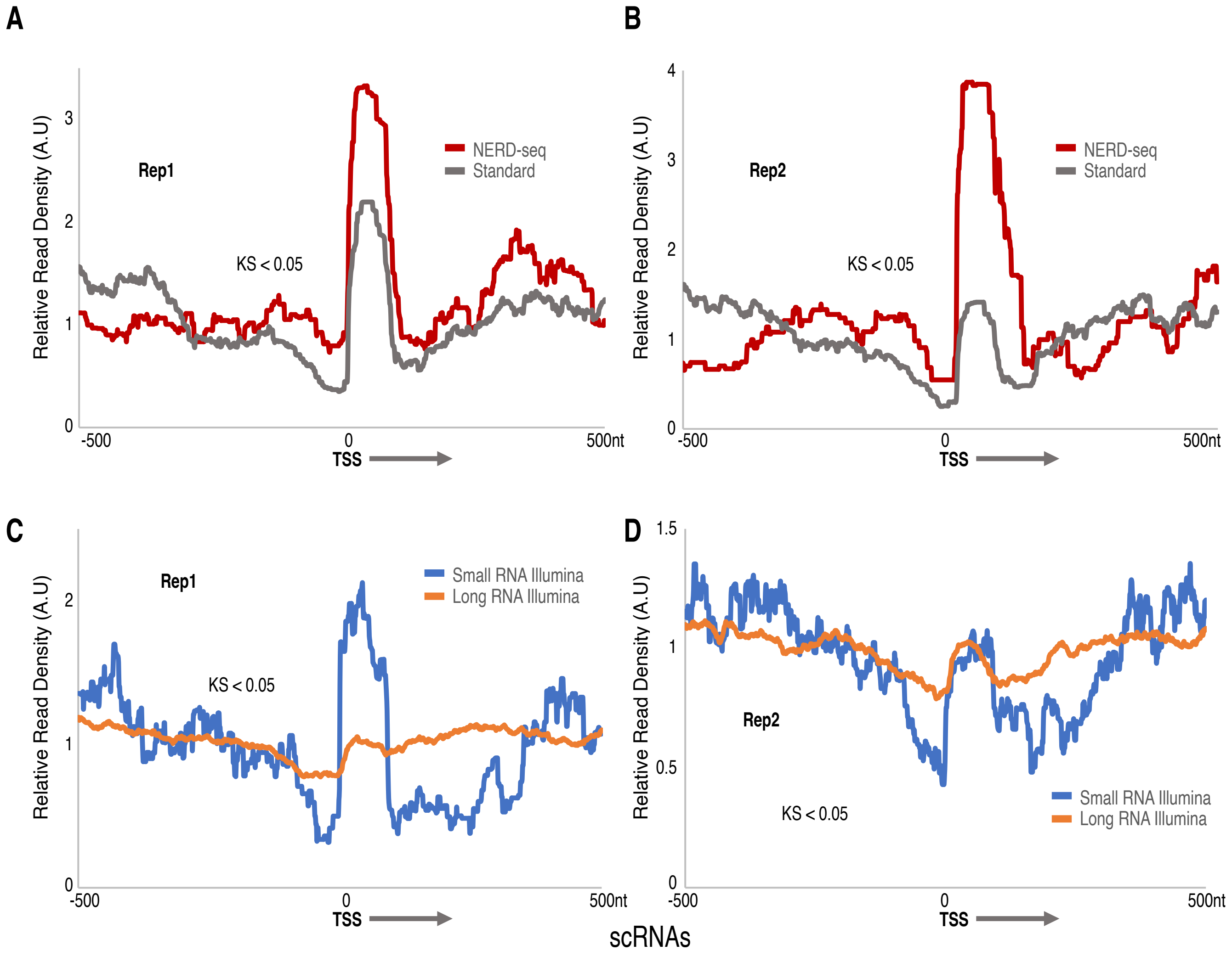

A
Rep1
NERD-seq
Standard
KS < 0.05
Relative Read Density (A.U)
TSS
-500
0
500nt
B
Rep2
NERD-seq
Standard
KS < 0.05
Relative Read Density (A.U)
TSS
C
Rep1
Small RNA Illumina
Long RNA Illumina
KS < 0.05
D
Rep2
Small RNA Illumina
Long RNA Illumina
KS < 0.05
scRNAs

Fig S14

Fig S14. NERD-seq reliably enriches scRNAs, similarly to Illumina short RNA libraries. Metagene plots depicting relative read density around the transcription start site (TSS) for gene family: snRNA. NERD-seq and standard libraries are compared in A-B with biological replicate 1 (A) and replicate 2 (B). X, Y axis and KS-test as in Figure 3. Similarly, Illumina long and short RNA libraries are compared for relative scRNA expression levels in C-D with the same biological replicates 1 (C) and 2 (D). X, Y axis and KS-test as in Figure 3.

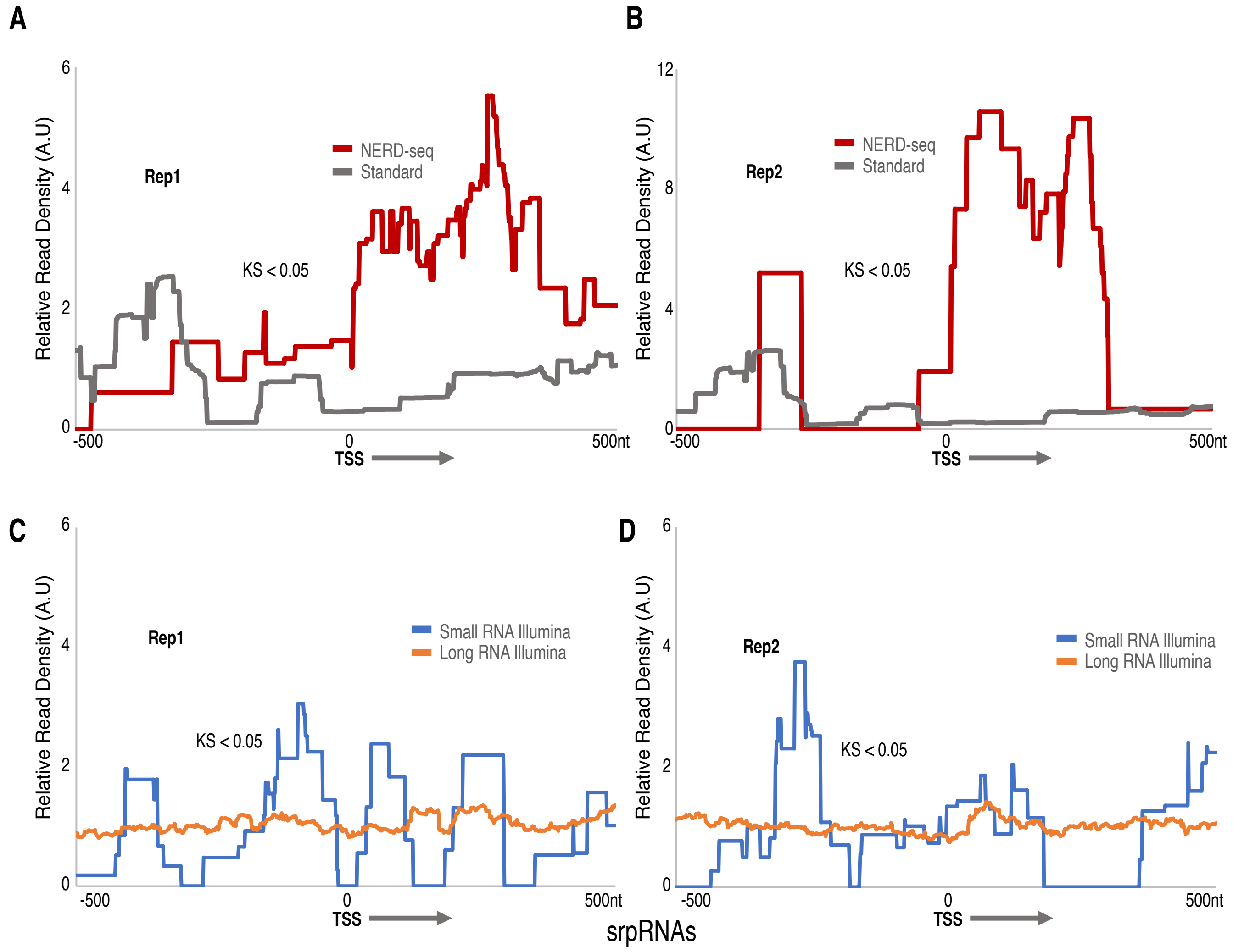
A
B
C
D
Relative Read Density (A.U)
Rep1
Rep2
NERD-seq
Standard
Small RNA Illumina
Long RNA Illumina
KS < 0.05
-500
0
500nt
TSS
srpRNAs

Fig S15

Fig S15. NERD-seq reliably enriches srpRNAs, similarly to Illumina short RNA libraries. Metagene plots depicting relative read density around the transcription start site (TSS) for gene family: srpRNA. NERD-seq and standard libraries are compared in A-B with biological replicate 1 (A) and replicate 2 (B). X, Y axis and KS-test as in Figure 3. Similarly, Illumina long and short RNA libraries are compared for relative srpRNA expression levels in C-D with the same biological replicates 1 (C) and 2 (D). X, Y axis and KS-test as in Figure 3.

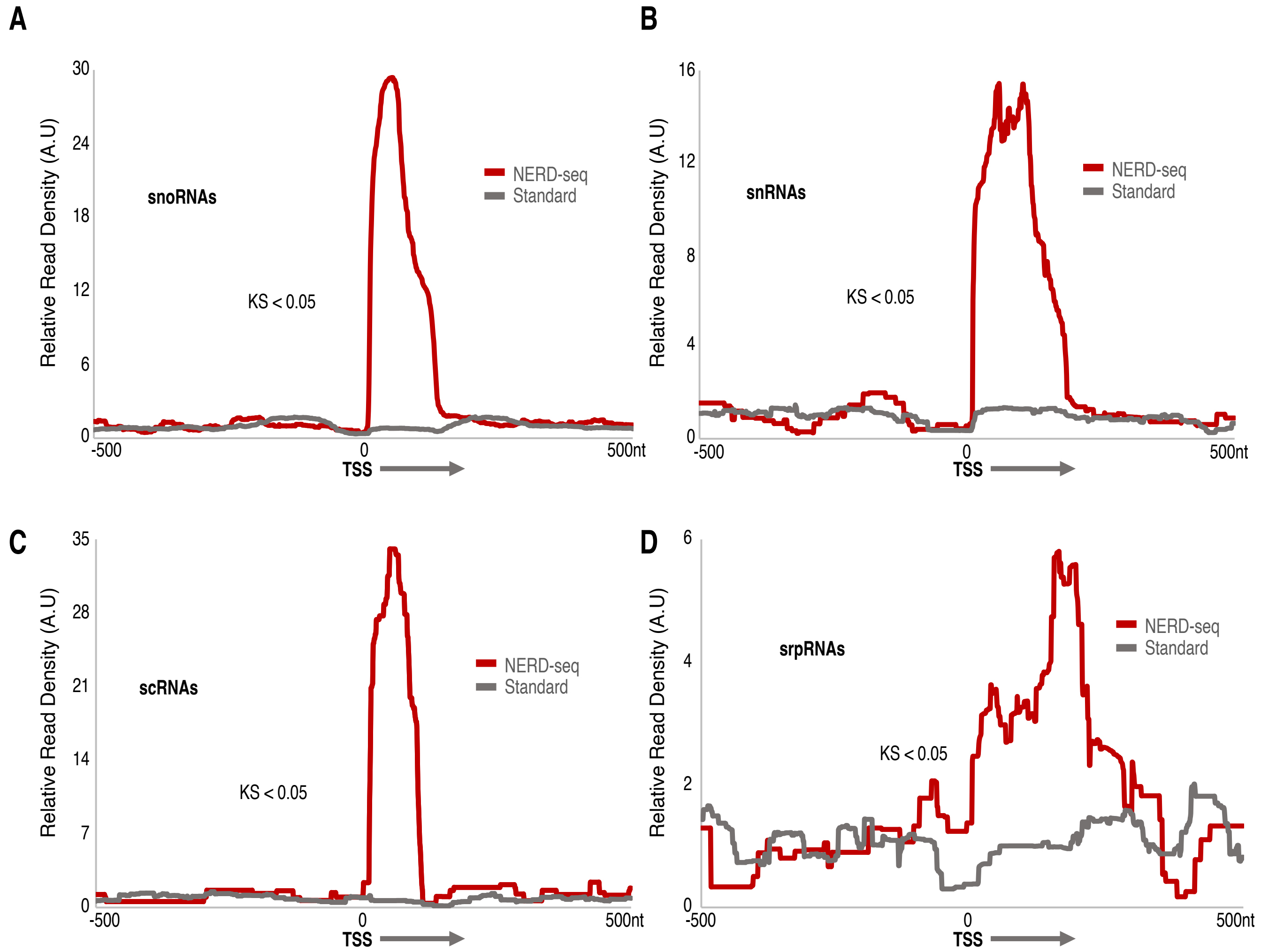
A
snoRNAs
KS < 0.05
NERD-seq
Standard
Relative Read Density (A.U)
30
24
18
12
6
0
-500
0
500nt
TSS
B
snRNAs
KS < 0.05
NERD-seq
Standard
Relative Read Density (A.U)
16
12
8
4
0
-500
0
500nt
TSS
C
scRNAs
KS < 0.05
NERD-seq
Standard
Relative Read Density (A.U)
35
28
21
14
7
0
-500
0
500nt
TSS
D
srpRNAs
KS < 0.05
NERD-seq
Standard
Relative Read Density (A.U)
6
4
2
0
-500
0
500nt
TSS

Fig S16

Fig S16. NERD-seq enriches ncRNAs in human cerebral cortex.

Metagene plots depicting relative read density around the transcription start site (TSS) for gene families: snoRNAs (A), snRNA (B), scRNAs (C), srpRNA (D), in human cerebral cortex derived RNAs. X, Y axis and KS-test as in Figure 3.

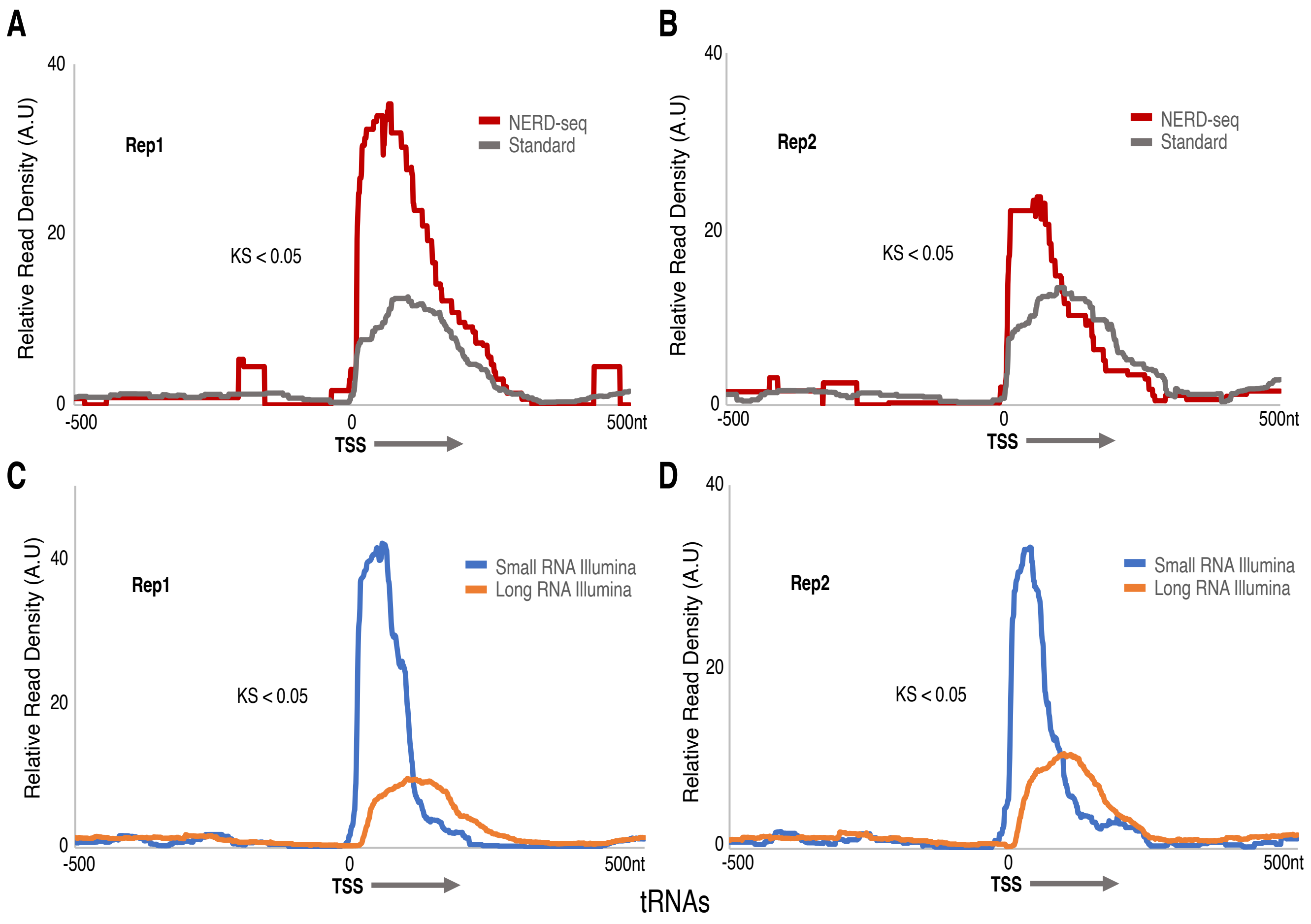
A
B
C
D
Relative Read Density (A.U)
Rep1
Rep2
KS < 0.05
NERD-seq
Standard
Small RNA Illumina
Long RNA Illumina
40
20
0
-500
0
500nt
TSS
tRNAs

Fig S17

Fig S17. NERD-seq reliably enriches tRNAs, similarly to Illumina short RNA libraries. Metagene plots depicting relative read density around the transcription start site (TSS) for gene family: tRNA. NERD-seq and standard libraries are compared in A-B with biological replicate 1 (A) and replicate 2 (B). X, Y axis and KS-test as in Figure 3. Similarly, Illumina long and short RNA libraries are compared for relative tRNA expression levels in C-D with the same biological replicates 1 (C) and 2 (D). X, Y axis and KS-test as in Figure 3.

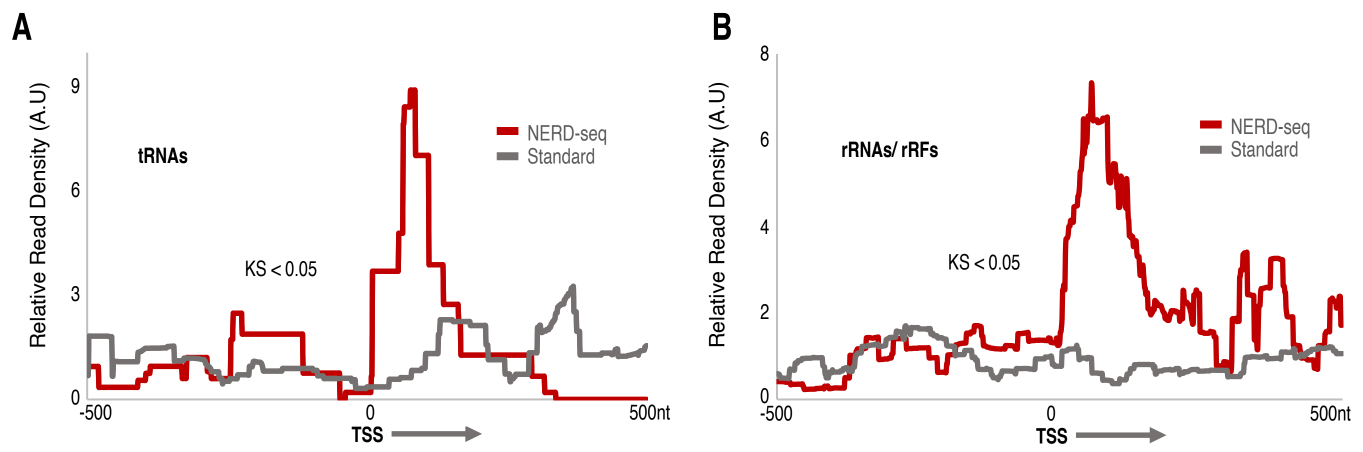
A
tRNAs
KS < 0.05
NERD-seq
Standard
Relative Read Density (A.U)
-500
0
500nt
TSS
B
rRNAs/ rRFs
KS < 0.05
NERD-seq
Standard
Relative Read Density (A.U)
-500
0
500nt
TSS

Fig S18

Fig S18. NERD-seq can detect tRNAs and rRNAs in human cerebral cortex.

Metagene plots depicting relative read density around the transcription start site (TSS) for gene families: tRNAs (A) and rRNAs (B), in human cerebral cortex derived RNAs. X, Y axis and KS-test as in Figure 3.

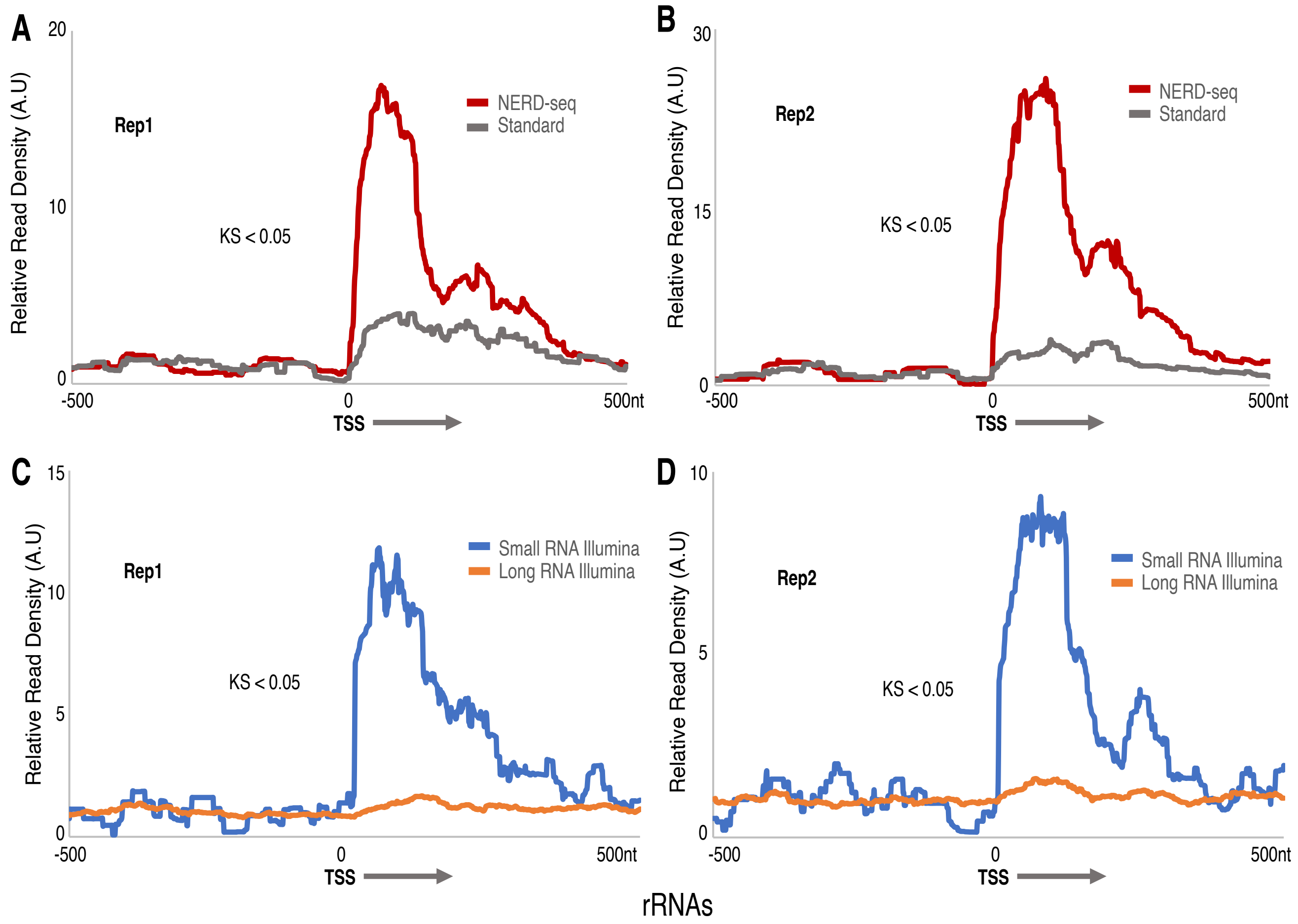

A
Rep1
KS < 0.05
NERD-seq
Standard
Relative Read Density (A.U)
20
10
0
-500
0
500nt
TSS
B
Rep2
KS < 0.05
NERD-seq
Standard
30
15
0
C
Rep1
KS < 0.05
Small RNA Illumina
Long RNA Illumina
15
10
5
D
Rep2
KS < 0.05
Small RNA Illumina
Long RNA Illumina
10
5
rRNAs

Fig S19

Fig S19. NERD-seq reliably enriches rRNAs, similarly to Illumina short RNA libraries. Metagene plots depicting relative read density around the transcription start site (TSS) for gene family**:** rRNA. NERD-seq and standard libraries are compared in A-B with biological replicate 1 (A) and replicate 2 (B). X, Y axis and KS-test as in Figure 3. Similarly, Illumina long and short RNA libraries are compared for relative rRNA expression levels in C-D with the same biological replicates 1 (C) and 2 (D). X, Y axis and KS-test as in Figure 3.

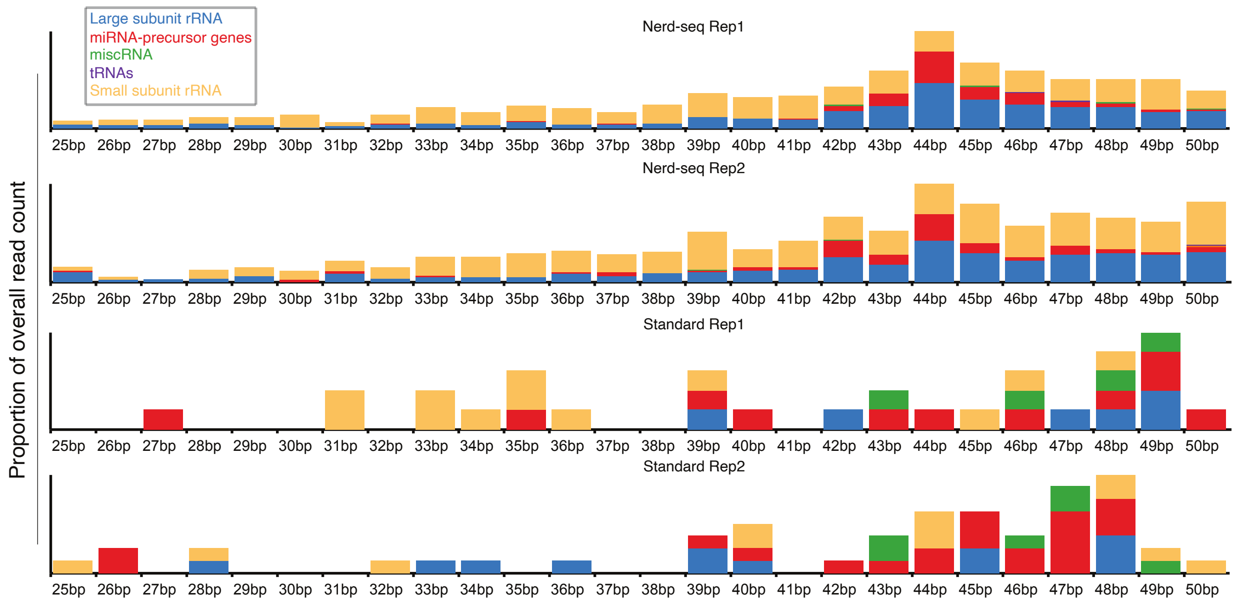
Large subunit rRNA
miRNA-precursor genes
miscRNA
tRNAs
Small subunit rRNA
Nerd-seq Rep1
Nerd-seq Rep2
Standard Rep1
Standard Rep2
Proportion of overall read count
25bp 26bp 27bp 28bp 29bp 30bp 31bp 32bp 33bp 34bp 35bp 36bp 37bp 38bp 39bp 40bp 41bp 42bp 43bp 44bp 45bp 46bp 47bp 48bp 49bp 50bp

Fig S20

Fig S20. Small RNAQC plot shows NERD-seq is more replicable than the standard approach in the 25-50bp range.

Small RNAQC plot produced using Seqmonk, demonstrating in the 25-50bp range distributions reads mapping to large subunit rRNAs, miRNA precursor genes, miscRNA, tRNAs and small subunit rRNAs. For each sequencing type, two biological replicates are depicted.

A

B

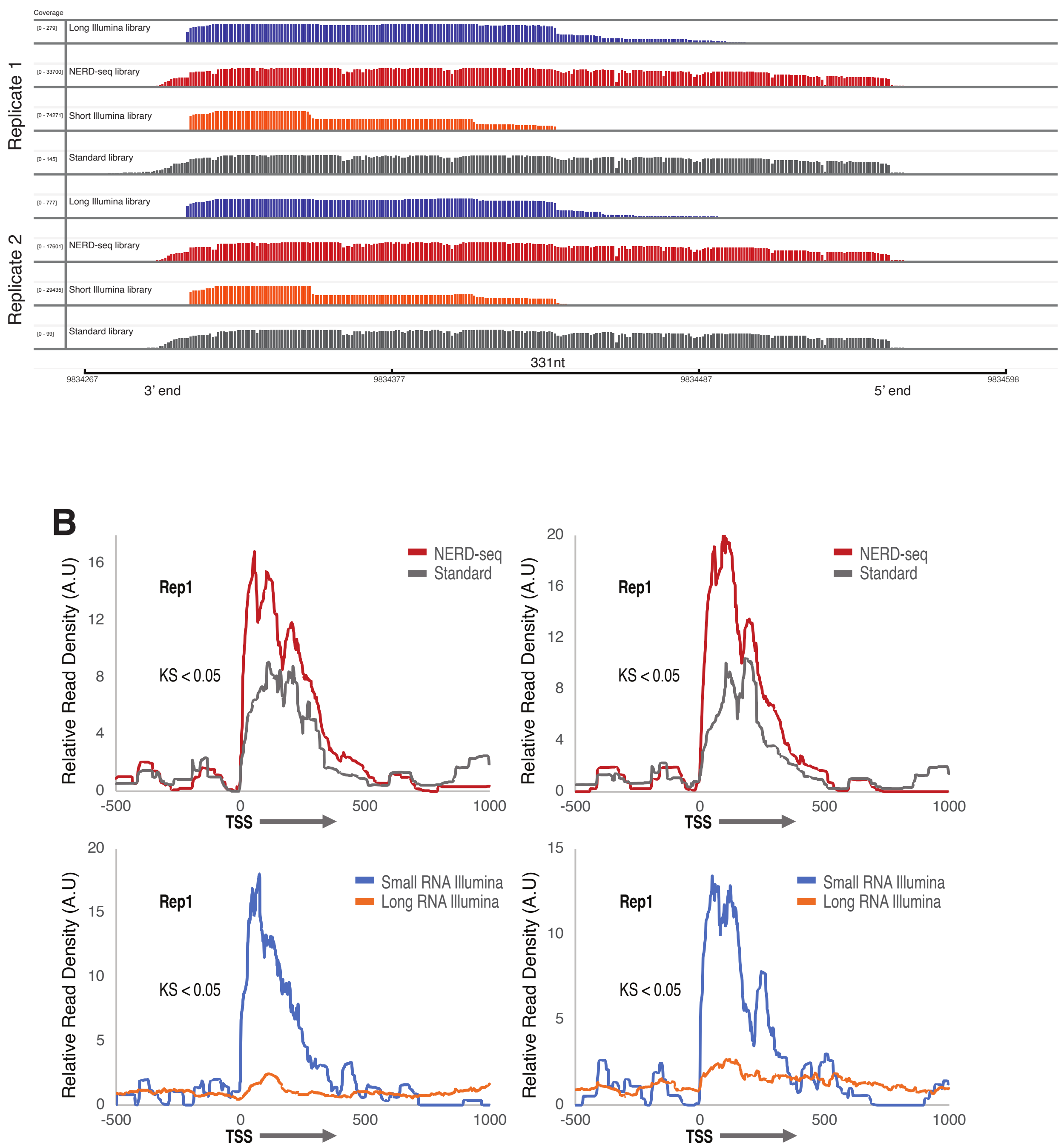

Coverage
Replicate 1
Long Illumina library
NERD-seq library
Short Illumina library
Standard library
Replicate 2
Long Illumina library
NERD-seq library
Short Illumina library
Standard library
331nt
9834267
9834377
9834487
9834598
3' end
5' end
Relative Read Density (A.U)
NERD-seq
Standard
Rep1
KS < 0.05
TSS
-500
0
500
1000
Small RNA Illumina
Long RNA Illumina

Fig S21

Fig S21. LSU-rRNA_HSA reads are enriched in the mouse hippocampus samples.

(A) IGV produced coverage plots between Chr13: 9,834,267-9834598. Illumina libraries (long and short) and Nanopore libraries (NERD and standard) are depicted in replicates of two. Coverage settings are as default in IGV and are represented in the leftmost label for each sample.

(B) Metagene plots depicting relative read density around the transcription start site (TSS) for repeat masker gene family: LSU-rRNA_HSA . NERD-seq and standard libraries are compared in A-B with biological replicate 1 (top left) and replicate 2 (top right). X, Y axis and KS-test as in Figure 3. Similarly, Illumina long and short RNA libraries are compared for relative LSU-rRNA_HSA expression levels with the same biological replicates 1 (bottom left) and 2 (bottom right). X, Y axis and KS-test as in Figure 3.

```
#=======================================
#
# Aligned_sequences: 2
# 1: BK000964.3
# 2: NERD
# Matrix: EDNAFULL
# Gap_penalty: 10.0
# Extend_penalty: 0.5
#
# Length: 259
# Identity:     257/259 (99.2%)
# Similarity:   257/259 (99.2%)
# Gaps:           1/259 ( 0.4%)
# Score: 1271.0
#
#
#=======================================

BK000964.3     12580 TCGTCCCCGCGGCGGCGCGGGGTCTCCCCCCGCCGGGCGTCGGGACCGGG  12629
                     ||||||||||||||||||||||||||||||||||||||||||||||||||
NERD               7 TCGTCCCCGCGGCGGCGCGGGGTCTCCCCCCGCCGGGCGTCGGGACCGGG     56

BK000964.3     12630 GTCCGGTGCGGAGAGCCGTTCGTCTTGGGAAACGGGGTGCGGCCGGAAAG  12679
                     ||||||||||||||||||||||||||||||||||||||||||||||||||
NERD              57 GTCCGGTGCGGAGAGCCGTTCGTCTTGGGAAACGGGGTGCGGCCGGAAAG    106

BK000964.3     12680 GGGGCCGCCCTCTCGCCCGTCACGTTGAACGCACGTTCGTGTGGAACCTG  12729
                     ||||||||||||||||||||||||||||||||||||||||||||||||||
NERD             107 GGGGCCGCCCTCTCGCCCGTCACGTTGAACGCACGTTCGTGTGGAACCTG    156

BK000964.3     12730 GCGCTAAACCATTCGTAGACGACCTGCTTCTGGGTCGGGGTTTCGTACGT  12779
                     |||||||||||||||||||||||||||||||||||| ||||||||||||.
NERD             157 GCGCTAAACCATTCGTAGACGACCTGCTTCTGGGTC-GGGTTTCGTACGC    205

BK000964.3     12780 AGCAGAGCAGCTCCCTCGCTGCGATCTATTGAAAGTCAGCCCTCGACACA  12829
                     ||||||||||||||||||||||||||||||||||||||||||||||||||
NERD             206 AGCAGAGCAGCTCCCTCGCTGCGATCTATTGAAAGTCAGCCCTCGACACA    255

BK000964.3     12830 AGGGTTTGT  12838
                     |||||||||
NERD             256 AGGGTTTGT    264


#---------------------------------------
```

Fig S22

Fig S22. L1Md_T mapped reads (mm10 – chr13: 9,832,020-9,838,665) in NERD-seq samples aligns with high similarity to the rDNA repeating gene (Genbank: BK000964.3).

Alignment produced using pairwise local aligner (Water-EMBOSS) in the EBI server (https://www.ebi.ac.uk/Tools/psa/emboss_water/). Query read was extracted from IGV as a consensus sequence with unassigned reads converted to reference identity.

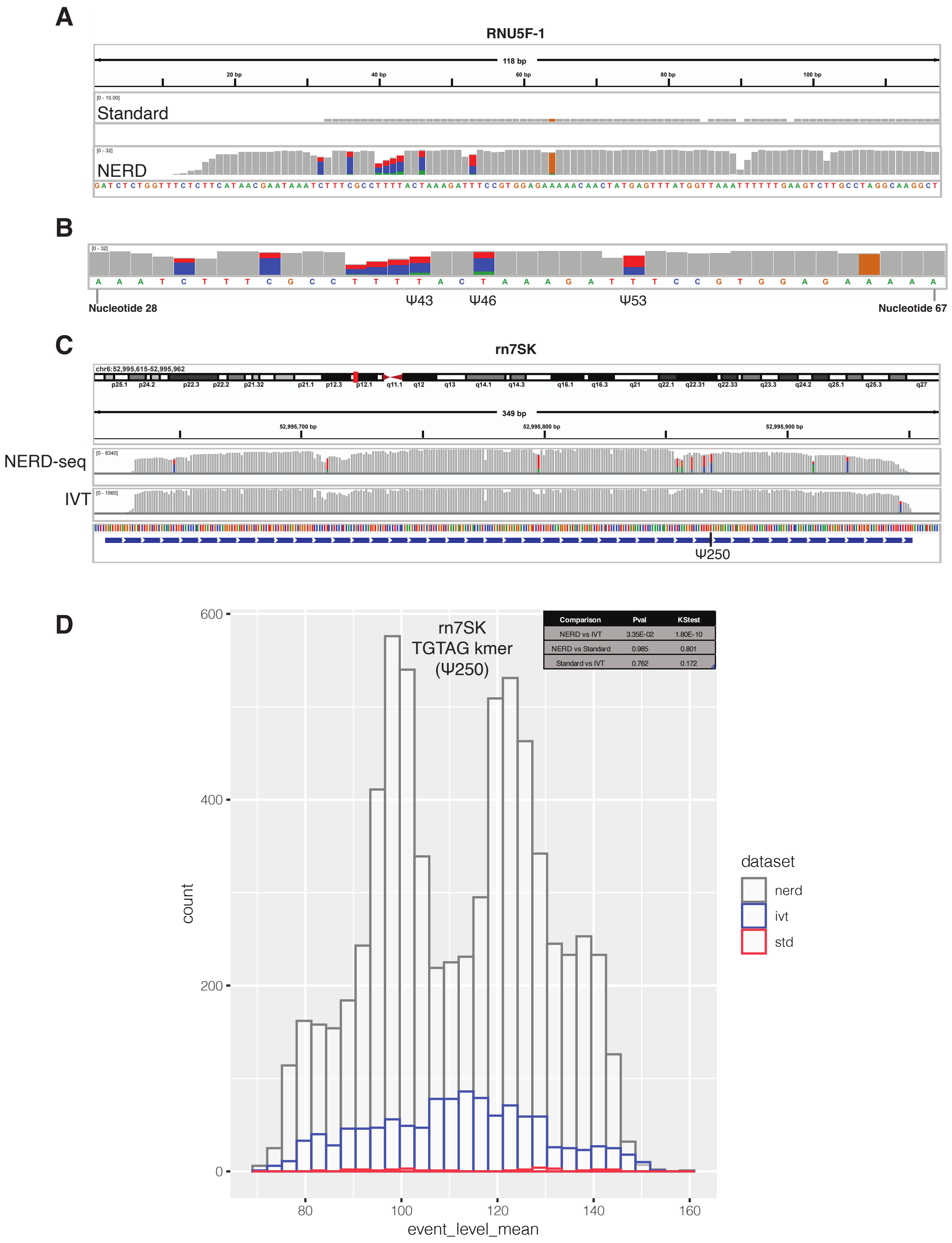
A
RNU5F-1
118 bp
20 bp
40 bp
60 bp
80 bp
100 bp
Standard
NERD
B
Nucleotide 28
Ψ43
Ψ46
Ψ53
Nucleotide 67
C
rn7SK
chr6:52,995,615-52,995,962
349 bp
52,995,700 bp
52,995,800 bp
52,995,900 bp
NERD-seq
IVT
Ψ250
D
rn7SK
TGTAG kmer
(Ψ250)
Comparison | Pval | KStest
NERD vs IVT | 3.35E-02 | 1.80E-10
NERD vs Standard | 0.985 | 0.801
Standard vs IVT | 0.762 | 0.172
count
event_level_mean
0
200
400
600
80
100
120
140
160
dataset
nerd
ivt
std

Fig S23

Fig S23. Known pseudouridine sites in ncRNAs are detectable using NERD-seq.

(A) IGV view of the RNU5F-1 genes from human cerebral cortex samples mapped against snRNA transcripts using minimap2 map-ont -ax k5 parameters.

(B) Zoomed in IGV view of RNU5f-1 gene on the NERD-seq sample within nucleotides 28-67. Coverage threshold for genomic to read mismatches are default and T to C mismatches are depicted as a genomic T with a blue and red bar. Known pseudouridine sites in snRNAs determined in Carlile *et al* 2014 (78) are depicted below the coverage plot at nucleotides 43, 46 and 53.

(C) IGV view of the rn7SK gene from human cerebral cortex samples mapped against the hg38 genome. Coordinates Chr6: 52,995,615-52,995,962 are depicted. Conserved pseudouridine 250 is annotated on a T to C mismatch in NERD-seq compared to a rn7SK IVT sample.

(D) Histogram depicting the distributions of the TGTAG kmer signal levels in rn7SK reads. Kmer signal is generated using Nanopolish eventalign and a table of Ttests and KStests are depicted in the top right corner. nerd depicts reads from the NERD-seq dataset, ivt depicts reads from the rn7SK IVT dataset and std depicts reads from the standard dataset.

Fig S23 demonstrates the NERD-seq protocol can produce the read coverage necessary to assess epitranscriptomic signatures and in the case of RNAs like RNU5f-1, NERD-seq is much more suitable an approach for studying epitranscriptomic markers than the standard approach.

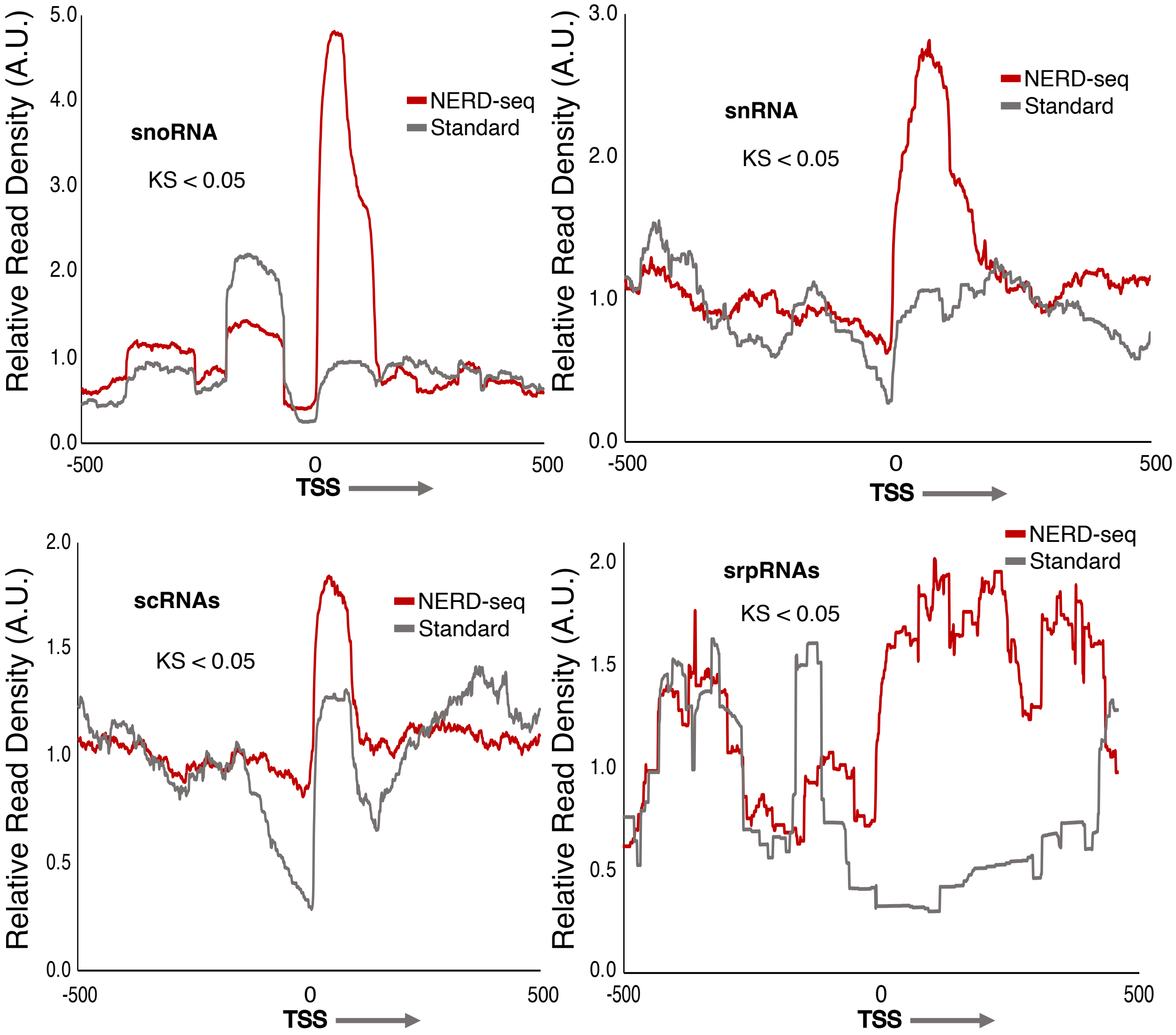
snoRNA
KS < 0.05
NERD-seq
Standard
Relative Read Density (A.U.)
5.0
4.0
3.0
2.0
1.0
0.0
-500
0
500
TSS
snRNA
KS < 0.05
3.0
2.0
1.0
0.0
scRNAs
2.0
1.5
0.5
srpRNAs

Fig S24

Fig S24. NERD-seq replicates patterns of enrichment with SQK_RNA004 Nanopore chemistry.

Metagene plots produced on data from RNA sequencing using the standard and a modified NERD-seq approach with the SQK_RNA004 direct RNA sequencing library chemistry.

(A) Relative read density distribution around the Transcription Start Site (TSS) of snoRNAs for external standard and NERD-seq. X, Y axis and KS-test as in Figure 3.

(B) Relative read density distribution around the Transcription Start Site (TSS) of snRNAs.

(C) Relative read density distribution around the Transcription Start Site (TSS) of scRNAs.

(D) Relative read density distribution around the Transcription Start Site (TSS) of srpRNA.
