# Supplementary material for: NERD-seq: a novel approach of Nanopore direct RNA sequencing that expands representation of non-coding RNAs
Source: Genome Biol. 2024 Aug 28;25:233. doi: 10.1186/s13059-024-03375-8 (PMC11351768; doi:10.1186/s13059-024-03375-8)
Supplement: Supplementary file 2 — Additional file 2: Includes a supplementary table featuring locations from consensus sequence of sequence present in L1Md_t through BLAT. [file 13059_2024_3375_MOESM2_ESM.pdf]

NERD-seq: A novel approach of Nanopore direct RNA sequencing that expands representation of non-coding RNAs

Luke Saville, Li Wu, Jemaneh Habtewold, Babita Gollen, Liam Mitchell, Matthew Stuart-Edwards, Travis Haight, Majid Mohajerani, Athanasios Zovoilis

Additional file 2

Supplementary Table

Table S1. BLAT table featuring locations from consensus sequence of sequence present in L1Md_t extracted from IGV viewer of NERD-seq replicate 1, within the coordinates: chr13: 9,832,020-9,838,665.

Chr	start	end	strand	score	match	mismatch
13	9834298	9834557	-	652	233	13
1	167340220	167340467	-	572	204	7
8	85111857	85112104	-	550	201	11
16	11144092	11144314	+	550	195	8
9	118674948	118675164	-	516	185	9
1	193632703	193632941	+	507	189	13
6	8728789	8729016	-	501	187	16
9	56223571	56223771	-	476	172	10
4	139338271	139338445	+	406	153	14
2	73867898	73868038	+	356	122	3
6	87997227	87997574	-	313	112	6
18	7030135	7030278	+	310	115	10
17	30134479	30134618	+	286	109	13
4	136538181	136538304	-	276	100	6
1	136365040	136365187	+	276	107	11
9	105819308	105819376	-	190	65	1
X	12801857	12801918	-	147	55	5
11	79389291	79389322	-	76	28	3
6	87997258	87997279	-	64	21	0
4	134213150	134213170	-	61	20	0
