# Supplementary material for: NERD-seq: a novel approach of Nanopore direct RNA sequencing that expands representation of non-coding RNAs
Source: Genome Biol. 2024 Aug 28;25:233. doi: 10.1186/s13059-024-03375-8 (PMC11351768; doi:10.1186/s13059-024-03375-8)
Supplement: Supplementary file 3 — Additional file 3: Peer review history. [file 13059_2024_3375_MOESM3_ESM.docx]

**Review history**

**First round of review**

**Reviewer 1**

Saville et al detail a new method, NERD-Seq, which extends nanopore direct RNA-Sequencing to beyond the poly(A) fraction. In principle, this allows the capture of additional RNA types in a single sequencing reaction, albeit at the expense of sequencing depth. However, the authors need to expand more on why this is useful, and what scenarios this would be used in. The rationale for many of their experimental choices are not explained/contextualized appropriately. The manuscript also suffers from excessive jargon and grammatical errors.

**Major comments:**

1: To my understanding, the primary purpose of NERD-Seq is to allow the sequencing of the non-adenylated fraction of RNAs within sample with a size filter step designed to exclude ncRNAs larger than ~200 nt. While this allow for subsequent polyadenylation of the small fraction, it remains quite likely that larger non-adenylated non-rRNAs are being missed by the protocol. It is also not clear why NERD-Seq should be considered the optimal strategy. The authors should show that they have considered/evaluated other strategies such as:
- Depletion/Seperation of the poly(A) fraction prior to size selection performed on non-adenylated fraction.
- Targeted degradation of the rRNA fraction using rRNA depletion kits.

2: Related to this, the authors note that NERD-Seq is designed to allow the sequencing of both adenylated and (short) non-adenylated fractions of RNAs within a study. What is the actual value of this versus, say, simply targeting the short non-adenylated fraction directly?

3: The manuscript in general is written in a somewhat subversive style, seemingly focused on highlighting the 'failings' of the standard ONT protocol. This is somewhat disingenuous as these are not 'failings' per se given the design objective of the standard DRS protocol (i.e. to capture and sequence the poly(A) fraction of RNAs) works well. The authors would be better off highlighting the situations (i.e. study questions) where NERD-Seq would provide a measurable benefit over the standard DRS strategy.

4: The authors state that 1.5ug total RNA is used as input for NERD-Seq but how much input (polyA RNA) actually goes into the short- and long-fraction parts of the DRS protocol? This is important to know.

5: The authors show that NERD-Seq performs well on a tissue that is generally enriched in non-coding RNA activity but without the inclusion of biology replicates or tissue samples from other sources, it is impossible to assess (1) the reproducibility/robustness of the methodology and (2) whether NERD-Seq would produce useable data from other tissues with lower non-coding RNA activity. These experiments are required.

6: Several figures are poorly explained. For instance, Does Figure 2A show data only from the short RNA fraction? If not then this suggests incredibly high levels of mRNA degradation in the 'standard' ONT fraction - far beyond what is seen in other studies. The legends for all figures should be far more specific and detailed.

**Minor quibbles:**

1: Please refrain from using 'next-generation' in a sequencing context (abstract, introduction). Having a 'new' next-generation sequencing is confusing in regard to the old 'next generation' sequencing.

2: Introduction should acknowledge that targeted sequencing of (individual) non-adenylated RNAs is possible (i.e. there is an ONT protocol for this).

3: Several figure elements not referenced in the text appropriately (e.g. red bars in Figure 2A).

4: The authors mention profiling of the epitranscriptome several times in the introduction and discussion but do not include any work geared toward looking for RNA modifications. Indeed it is entirely unclear whether NERD-Seq produces the depth of sequencing (on non-adenylated RNAs) required for current RNA modification detection tools.

NERD-Seq presents a modified method for DRS of both short non-adenylated and the standard adenylated fraction of RNAs within a sample. This utility of this approach appears rather niche and it is hard to determine situations in which this approach would be generally useful versus many of the existing (Illumina-based) approaches. The burden to demonstrate that NERD-Seq is more than an incremental improvement to existing approaches lies with the authors.

**Reviewer 2**

In this manuscript Saville et al. present NERD-seq, a novel method to enrich and directly sequence non-coding RNA using the Oxford Nanopore Technology (ONT). The technique is based on the addition of a poly-A tail to small non-coding RNAs which enables their sequencing by a conventional ONT direct RNA-seq protocol using a poly(T)-tethering adaptor. This technique has great potential, as ONT direct RNA sequencing can be used to detect RNA modifications. Furthermore, non-coding RNAs are known to harbor a lot of these modifications and that they are important to their function. In addition, non-coding RNAs aren't generally poly-adenylated and therefore are underrepresented when using standard ONT direct RNA-seq. The authors have shown that using NERD-seq, they are able to significantly improve the number of reads on several subsets of non-coding RNAs : tRNAs, snoRNAs, snRNAs, scRNAs and rRFs. There are however some issues that need to be addressed before the manuscript is suitable to publication.

**Major Comments:**

• Data on RNA modifications present in the sequenced non-coding RNA would greatly improve the impact of the manuscript, as the method is presented as read tool to study RNA modifications. If the authors can't include these data, they should at least explain why in the discussion section.

• The authors did not discuss possible biases of their method. For example, is the poly-A tail ligation efficiency affected by sequence and or structural differences between RNA? Is there detectable differences in the efficiency of sequencing small RNA with structured 3'end when compared to mRNA? Are the proportions of reads obtained per non-coding RNA sub-category and RNA rank supported by other biochemical data (e.g. Northern blots, primer extension, RT-qPCR?

• It is essential to include external size and sequence markers spike-in to directly evaluate the quality and fidelity of the newly develop sequencing technique.

• The author compared NERD-seq to ONT standard direct RNA-seq but did not compare it with other methods that are currently used for non-coding RNA sequencing (e.g. Illumina based sequencing, TGIRTseq). There is a need for side by side comparison at least by using data available in the literature if not experimentally.

**Minor Comments:**

• Page 5 line 3, NERD-seq is misspelled.

• Throughout this section µL and µg are written as uL and ug.

• Several temperatures are missing the o symbol between the number and C.

We recommend that all these issues should be addressed before publication.

This manuscript has the potential of having major significance to researchers in the field of transcriptomics if the author demonstrated that their newly sequencing techniques could indeed improve RNA detection beyond the current techniques. Unfortunately, the manuscript only show the capacity of the technique to detect non-coding RNA but fall short from providing strong arguments supporting the fidelity, accuracy and coverage of the new technique. The use of Nanopore sequencing is not unique and was used in the past for sequencing non-coding RNA. What is needed now is a proof of the increased accuracy or utility of the new technique to justify yet another publication about Nanopore sequencing paper. The manuscript would potentially be of interest to researcher working on different type of non-coding RNA and transcriptomics.

I am in the field of non-coding RNA sequencing and functional analysis and very familiar with this approach.

**Authors’ response to reviewers**

## Reviewer 1

*Reviewer #1 (Evidence, reproducibility and clarity (Required)):*

***Summary:***

**This reviewer generally remarks that**: “*Saville et al detail a new method, NERD-Seq, which extends nanopore direct RNA-Sequencing to beyond the poly(A) fraction. In principle, this allows the capture of additional RNA types in a single sequencing reaction, albeit at the expense of sequencing depth. However, the authors need to expand more on why this is useful, and what scenarios this would be used in. The rationale for many of their experimental choices are not explained/contextualized appropriately. The manuscript also suffers from excessive jargon and grammatical errors.”*

We appreciate these comments made by the reviewer. We now expand more on why and how NERD-seq is useful, and what scenarios this could be used in. In particular:

In the introduction, we now denote that a significant number of the most well described RNA modification positions are located in classes of RNAs such as tRNAs, snoRNAs and other ncRNAs like 7SK RNA. In the results section, we build on the second reviewer’s suggestion that “*This technique has great potential, as ONT direct RNA sequencing can be used to detect RNA modifications…*”, and we first show in revised Figures 3-5 that the standard approach misses the above classes of RNAs. Subsequently, in two new figures (Fig. 7 and Suppl.Fig. S23) we present an example of the ability of NERD-seq to decipher known RNA modifications in a well-studied ncRNA, 7SK, compared with the inability of the standard approach to do so. Widely studied RNAs such as 7SK have the potential and thus often serve as controls for known positions of certain RNA modifications when validating them for novel positions in other RNAs. This however means that the application of the standard approach, which is not able to detect RNAs and thus their modifications for RNAs such as 7SK, makes it difficult to use them as controls. In other words, the ability to perform genome wide studies of RNA modifications using the direct RNA-seq approach relies on the ability to be able to confirm at the same time these findings in already known control positions located in the above classes of RNAs. If these known controls such as short ncRNAs are missing, as we show that it is the case with standard RNA-seq, it is difficult to perform such genome wide epitranscriptome studies. Thus, the usefulness of our approach is not only that it can sequence certain classes of shorter RNAs beyond mRNAs, but that it can do this without affecting the ability to study mRNAs simultaneously or use targeted sequencing adapters. This is now discussed in the text. We have also tried to avoid the excessive use of jargon language and correct any grammatical errors.

***Major comments:***

**Point 1. The reviewer mentions:** *“To my understanding, the primary purpose of NERD-Seq is to allow the sequencing of the non-adenylated fraction of RNAs within sample with a size filter step designed to exclude ncRNAs larger than ~200 nt. While this allow for subsequent polyadenylation of the small fraction, it remains quite likely that larger non-adenylated non-rRNAs are being missed by the protocol. It is also not clear why NERD-Seq should be considered the optimal strategy. The authors should show that they have considered/evaluated other strategies such as:*

- *Depletion/Seperation of the poly(A) fraction prior to size selection performed on non-adenylated fraction.*
- *Targeted degradation of the rRNA fraction using rRNA depletion kits.”*

We thank the reviewer for this comment. The primary purpose of NERD-seq is not only to allow the sequencing of the non-adenylated fraction of shorter RNAs but to do this while maintaining the ability to sequence longer polyadenylated RNAs. In particular, we are not sequencing only the short RNA fraction but also the longer RNA fraction of naturally polyadenylated RNAs. This includes both mRNAs and ncRNAs that are polyadenylated. We apologize that this may have not be presented clearly in our initial text, and we have now revised the respective results part (page 8, lines 13-14 and 30-31). As mentioned below, we now also provide evidence that our approach does not affect the ability to efficiently sequence mRNAs and decipher effectively their isoforms (new Suppl. Fig. S19). Only large non poly-A ncRNAs still evade our detection. Now, we discuss this limitation in the last paragraph of the discussion section (page 19, lines 12-20). We have now also evaluated other strategies such as those mentioned by the reviewer (depletion of rRNA, size selection using magnetic beads, different enzymes) and in the new Suppl. Figure S5 we show that they were suboptimal in providing sufficient reads that pass the quality thresholds in base calling compared to our NERD-seq approach.

To sum up, we certainly don’t claim to be able to capture longer ncRNAs, due to their lack of polyadenylated tails. We show though that while the ability to capture simultaneously all reads under the current Nanopore sequencing protocols may be unattainable, the NERD-seq methodology demonstrates a useful addition to the Nanopore sequencing repertoire due to its ability, in addition to polyadenylated transcripts, to simultaneously capture multiple classes of short ncRNAs, hitherto until now only achievable one transcript at a time with custom adaptor ligation.

**Point 2. The reviewer mentions that** *“Related to this, the authors note that NERD-Seq is designed to allow the sequencing of both adenylated and (short) non-adenylated fractions of RNAs within a study. What is the actual value of this versus, say, simply targeting the short non-adenylated fraction directly? ”*

Please see our response to the general comment by this reviewer and the new figures Fig. 7 and Suppl. Fig. S23 and the completely new results section at pages 15 and 16 about examples on how NERD-seq expands the study of epitranscriptomic signatures to additional RNA classes while maintaining the ability to efficiently assess the protein coding transcriptome (new Suppl. Fig. S19).

**Point 3. The reviewer notes that** *“The manuscript in general is written in a somewhat subversive style, seemingly focused on highlighting the 'failings' of the standard ONT protocol. This is somewhat disingenuous as these are not 'failings' per se given the design objective of the standard DRS protocol (i.e. to capture and sequence the poly(A) fraction of RNAs) works well. The authors would be better off highlighting the situations (i.e. study questions) where NERD-Seq would provide a measurable benefit over the standard DRS strategy. ”*

We apologize if this was indicated in our initial submission, as this was not our intention. We now focus on highlighting what NERD-seq can do, and this is reflected in the changes we have made in the introduction and discussion section.

**Point 4. The reviewer asks** *“The authors state that 1.5ug total RNA is used as input for NERD-Seq but how much input (poly-A RNA) actually goes into the short- and long-fraction parts of the DRS protocol? This is important to know.* ”

We have now included data in the manuscript for polyadenylation signals in the reads themselves (new Suppl. Fig S5F) and view it as a useful addition to the manuscript as it seems to show there is little difference in the relationship of length of transcript and polyadenylation tail length between the two methods.

RNA RIN values differ across samples so within different samples the portions of poly-A RNA, non poly- A RNA and former poly-A RNA that has lost the poly-A due to degradation may also vary. This however has not been shown to affect the reproducibility of the standard direct RNA-seq methodology, so it should not affect also that of NERD-seq. Nevertheless, we searched the literature for a well characterized methodology for quantifying this further and found little on poly(A) specific quantification approaches that we could use. In fact, poly(A) selection has also been shown to have variable efficiency and could thus produce inaccurate measurements that would make it difficult for us to assess explicitly the exact portions of poly-A vs non poly-A portions. Thus, we feel that in the absence of a well characterized methodology to make these quantifications, developing a new one may have been beyond the scope of this manuscript.

**Point 5. The reviewer recommends:** *“The authors show that NERD-Seq performs well on a tissue that is generally enriched in non-coding RNA activity but without the inclusion of biology replicates or tissue samples from other sources, it is impossible to assess (1) the reproducibility/robustness of the methodology and (2) whether NERD-Seq would produce useable data from other tissues with lower non-coding RNA activity. These experiments are required. ”*

We thank the reviewer for this suggestion and we have now included an additional biological replicate for the mouse tissue and data from another tissue in a separate organism (5-person pool source human cerebral cortex RNA).

**Point 6. The reviewer notes:** *“Several figures are poorly explained. For instance, Does Figure 2A show data only from the short RNA fraction? If not then this suggests incredibly high levels of mRNA degradation in the 'standard' ONT fraction - far beyond what is seen in other studies. The legends for all figures should be far more specific and detailed.”*

We have now replaced figure 2A with a different mapping strategy and a whole sample assessment of mapped reads lengths. We have also edited the figure legends to include more information.

## The reviewer made also the following comments:

*Minor comments:*

*“: Please refrain from using 'next-generation' in a sequencing context (abstract, introduction). Having a 'new' next-generation sequencing is confusing in regard to the old 'next generation' sequencing. ”*

The instances mentioned have been removed as per the reviewer’s suggestion.

*“Introduction should acknowledge that targeted sequencing of (individual) non-adenylated RNAs is possible (i.e. there is an ONT protocol for this).”*

We now include mention of this in the introduction, results and discussion.

*“Several figure elements not referenced in the text appropriately (e.g. red bars in Figure 2A). ”*

We have now addressed this and thank the reviewer for making us aware of this.

*“The authors mention profiling of the epitranscriptome several times in the introduction and discussion but do not include any work geared toward looking for RNA modifications. Indeed it is entirely unclear whether NERD-Seq produces the depth of sequencing (on non-adenylated RNAs) required for current RNA modification detection tools. ”*

We have now included a new main figure (Fig. 7) and a new supplementary figure (Suppl. Fig. S23) which profile elements of the epitranscriptome and show that indeed NERD-seq produces the depth of sequencing necessary for RNA modification detection.

*Reviewer 1’s summary:*

*“NERD-Seq presents a modified method for DRS of both short non-adenylated and the standard adenylated fraction of RNAs within a sample. This utility of this approach appears rather niche and it is hard to determine situations in which this approach would be generally useful versus many of the existing (Illumina-based) approaches. The burden to demonstrate that NERD-Seq is more than an incremental improvement to existing approaches lies with the authors. ”*

We appreciate the reviewer’s comments. While we agree with them that for various applications (such as differential gene expression, quantification of lowly expressed genes) “*the existing (Illumina-based) approaches*” Illumina RNA-seq or short-RNA-seq would be more appropriate due to higher sequence yields, the main advantage of nanopore direct RNA-seq is the ability to identify RNA modifications directly at the same time (see new Figs 7 and S23). While we also agree that the field of high throughput sequencing epitranscriptomics is quite nascent, it is quickly gaining interest. Based on the reviewer’s suggestions we believe that we have now modified the manuscript substantially to provide justification for NERD-seq’s feasibility as a methodology to capture multiple classes of ncRNAs for the study of their sequence, quantity and their epitranscriptomic signatures.

We would also like to note that even as a BioRxiv preprint, our manuscript has received already a number of citations, including from an article published in Nature Biotechnology and feel this demonstrates interest in NERD-seq from the scientific community.

## Reviewer 2

*Reviewer #2 (Evidence, reproducibility and clarity (Required))*

**This reviewer generally remarks that** *“In this manuscript Saville et al. present NERD-seq, a novel method to enrich and directly sequence non-coding RNA using the Oxford Nanopore Technology (ONT). The technique is based on the addition of a poly-A tail to small non-coding RNAs which enables their sequencing by a conventional ONT direct RNA-seq protocol using a poly(T)-tethering adaptor. This technique has great potential, as ONT direct RNA sequencing can be used to detect RNA modifications. Furthermore, non-coding RNAs are known to harbor a lot of these modifications and that they are important to their function. In addition, non-coding RNAs aren't generally poly-adenylated and therefore are underrepresented when using standard ONT direct RNA-seq. The authors have shown that using NERD-seq, they are able to significantly improve the number of reads on several subsets of non-coding RNAs : tRNAs, snoRNAs, snRNAs, scRNAs and rRFs. There are however some issues that need to be addressed before the manuscript is suitable to publication.”*

We thank the reviewer for their encouraging comments.

*Major comments:*

**Point 1. The reviewer recommends that** *“Data on RNA modifications present in the sequenced non- coding RNA would greatly improve the impact of the manuscript, as the method is presented as read tool to study RNA modifications. If the authors can't include these data, they should at least explain why in the discussion section.”*

We have now included a new main figure (Fig.7) and a supplemental figure (Suppl. Fig. S23) which show the ability of NERD-seq to identify previously described RNA modifications and its superiority in the case of non-coding RNAs such as 7SK. We thank the reviewer for this recommendation that significantly improved our manuscript better revealing the rationale and impact of our approach.

**Point 2. The reviewer remarks that** *“The authors did not discuss possible biases of their method. For example, is the poly-A tail ligation efficiency affected by sequence and or structural differences between RNA? Is there detectable differences in the efficiency of sequencing small RNA with structured 3'end when compared to mRNA? Are the proportions of reads obtained per non-coding RNA sub-category and RNA rank supported by other biochemical data (e.g. Northern blots, primer extension, RT-qPCR? ”*

We have included commentary in the discussion on these biases (page 19, lines 7-11). We also include a new supplementary figure (Suppl. Fig. S5F) regarding polyadenylation in which we see similar transcript length to polyadenylation tail length dynamics between the two methodologies.

**Point 3. The reviewer notes that** *“It is essential to include external size and sequence markers spike-in to directly evaluate the quality and fidelity of the newly develop sequencing technique. ”*

We have now repeated the sequencing, including sequencing with the well described RNA sequins mix B. We include a new supplementary figure with this data (Suppl. Fig. S10) that shows that NERD-seq does not differ from the standard approach regarding its ability to effectively evaluate the quantity and complexity of the transcripts present.

**Point 4. The reviewer mentions that** *“The author compared NERD-seq to ONT standard direct RNA-seq but did not compare it with other methods that are currently used for non-coding RNA sequencing (e.g. Illumina based sequencing, TGIRTseq). There is a need for side by side comparison at least by using data available in the literature if not experimentally. ”*

We now include a comparison with Illumina sequencing data for both long and short fractions from each biological replicate (Suppl.Figs S12-15,17,19 and 21). This data shows that NERD-seq combines the advantages of both standard and short RNA-seq with Illumina, enriching both long and short RNAs, while simultaneously as mentioned above, offering the benefits of direct RNA-seq. We feel this also demonstrates the utility of the unique chemistry of Nanopore sequencing where fragmenting of long RNAs is not required for sequencing, allowing the combination of long RNAs and short RNAs in the same library to reproduce what is essentially enriched in short RNA libraries as well as poly(A) sequencing libraries. Since the enzyme included in our initial submission is not anymore widely accessible, we also tested a series of other enzymes, including the commercial replacement for omniamp (the originally used enzyme), lavalamp, and other small tweaks to the library methodology, until finding the GSP SSD2.0 enzyme to be the most suitable for our research goals. The summary of our findings is in Figure S5. Initially, we considered TGIRT as a potential enzyme for the sequencing reaction because of its use for sequencing snoRNAs and other highly structured RNAs but because of its strand switching properties, we felt it would be poorly suited for a nanopore direct RNA sequencing approach.

## The reviewer made also the following comments/suggestions:

*Minor comments*

*“Page 5 line 3, NERD-seq is misspelled. ”*

This is now fixed.

*“Throughout this section µL and µg are written as uL and ug.”*

This is now fixed.

*“Several temperatures are missing the o symbol between the number and C.”*

This is now fixed.

*Final comments:*

*“This manuscript has the potential of having major significance to researchers in the field of transcriptomics if the author demonstrated that their newly sequencing techniques could indeed improve RNA detection beyond the current techniques. Unfortunately, the manuscript only show the capacity of the technique to detect non-coding RNA but fall short from providing strong arguments supporting the fidelity, accuracy and coverage of the new technique. The use of Nanopore sequencing is not unique and was used in the past for sequencing non-coding RNA. What is needed now is a proof of the increased accuracy or utility of the new technique to justify yet another publication about Nanopore sequencing paper. The manuscript would potentially be of interest to researcher working on different type of non-coding RNA and transcriptomics.*

*I am in the field of non-coding RNA sequencing and functional analysis and very familiar with this approach.”*

We thank the reviewer again for their encouraging comments and valid concerns. We agree that the initial manuscript fell short of providing evidence of its utility as an addition to the Nanopore sequencing repertoire. We hope the additional data we provide (rerunning with an additional enzyme, use of replicates, use of standard spike in controls, testing an additional organism and tissue, testing and comparing different enzymes and other sequencing platforms) provide the necessary assurances about reproducibility and accuracy, while the new data concerning identification of RNA modifications and detection of important RNA classes missed by the standard protocol provides the necessary assurances about the utility of our approach.

**Second round of review**

**Reviewer 1**

The authors have gone to great efforts to satisfy the critiques provided by the reviewers and I commend them for this. The manuscript is significantly stronger and it is clear that NERD-Seq has the potential to become a widely-used approach.

I have only two final points.

The first relates to the 'short fraction' of the RNA. Lucas et al recently developed a nanopore-based approach for sequencing mature tRNAs and as part of this work demonstrated that large numbers of RNAs < 150 nt are 'rejected' during sequencing (with the RNA002 chemistry) and are not recorded in the final dataset (PMID: 37024678). Presumably this applied to NERD-Seq as well.

The second point relates to the newly developed SQK-RNA004 nanopore chemistry and the discontinuation of the older SQK-RNA002 chemistry. My experience with the new chemistry suggests that NERD-Seq performance should not be negatively impacted and the new chemistry now allows capture of RNAs > 20nt in length.

While I would not ask the authors to perform further sequencing experiments at this point, I do think the above points would be valuable include in their discussion to highlight how 'future proof' the NERD-Seq approach is.

**Reviewer 2**

Reviewer 1 assessed the authors’ response to Reviewer 2 and was satisfied that their concerns were addressed.

**Authors’ response to reviewers**

The first point is now discussed extensively in page 16, lines 14-21. For the second point we now provide a new results section and also a supplementary figure, Fig S24, with sequencing using the new RNA chemistry and show that the ability of NERD-seq to detect short ncRNA classes remains unchanged. The methods section has also been updated accordingly and this is also now discussed in Discussion, page 16, lines 21-27.
